# Comparative genomics revealed adaptive admixture in *Cryptosporidium hominis* in Africa

**DOI:** 10.1099/mgen.0.000493

**Published:** 2020-12-23

**Authors:** Swapnil Tichkule, Aaron R. Jex, Cock van Oosterhout, Anna Rosa Sannella, Ralf Krumkamp, Cassandra Aldrich, Oumou Maiga-Ascofare, Denise Dekker, Maike Lamshöft, Joyce Mbwana, Njari Rakotozandrindrainy, Steffen Borrmann, Thorsten Thye, Kathrin Schuldt, Doris Winter, Peter G. Kremsner, Kwabena Oppong, Prince Manouana, Mirabeau Mbong, Samwel Gesase, Daniel T. R. Minja, Ivo Mueller, Melanie Bahlo, Johanna Nader, Jürgen May, Raphael Rakotozandrindrain, Ayola Akim Adegnika, John P. A. Lusingu, John Amuasi, Daniel Eibach, Simone Mario Caccio

**Affiliations:** ^1^​ Population Health and Immunity, Walter and Eliza Hall Institute of Medical Research, Melbourne, VIC, Australia; ^2^​ Faculty of Medicine, Dentistry and Health Sciences, University of Melbourne, Melbourne, VIC, Australia; ^3^​ Department of Medical Biology, University of Melbourne, Melbourne, Australia; ^4^​ Faculty of Veterinary and Agricultural Sciences, University of Melbourne, Melbourne, VIC, Australia; ^5^​ School of Environmental Sciences, University of East Anglia, Norwich Research Park, Norwich, UK; ^6^​ Department of Infectious Disease, Istituto Superiore di Sanità, Viale Regina Elena 299, 00161, Rome, Italy; ^7^​ Department of Infectious Disease Epidemiology, Bernhard Nocht Institute for Tropical Medicine Hamburg, Bernhard-Nocht-Strasse 74, 20359 Hamburg, Germany; ^8^​ German Center for Infection Research (DZIF), Hamburg-Lübeck-Borstel-Riem s, Germany; ^9^​ Division of Infectious Diseases and Tropical Medicine, University Hospital, LMU Munich, Munich 80802, Germany; ^10^​ Kumasi Centre for Collaborative Research in Tropical Medicine, College of Health Sciences, KNUST, Kumasi, Ghana; ^11^​ National Institute for Medical Research, Tanga Research Centre, Tanga, Tanzania; ^12^​ University of Antananarivo, BP 566, Antananarivo 101, Madagascar; ^13^​ Centre de Recherches Médicales de Lambaréné, BP 242 Lambaréné, Gabon; ^14^​ Institut für Tropenmedizin and German Center for Infection Research, partner site Tübingen, Universitätsklinikum, Wilhelmstraße 27, 72074 Tübingen, Germany; ^15^​ Department of Genetics and Bioinformatics, Division of Health Data and Digitalisation, Norwegian Institute of Public Health, Oslo, Norway

**Keywords:** *Cryptosporidium hominis*, whole-genome sequencing, population structure, recombination, genetic introgression, Africa

## Abstract

Cryptosporidiosis is a major cause of diarrhoeal illness among African children, and is associated with childhood mortality, malnutrition, cognitive development and growth retardation. *Cryptosporidium hominis* is the dominant pathogen in Africa, and genotyping at the glycoprotein 60 (*gp60*) gene has revealed a complex distribution of different subtypes across this continent. However, a comprehensive exploration of the metapopulation structure and evolution based on whole-genome data has yet to be performed. Here, we sequenced and analysed the genomes of 26 *C*. *hominis* isolates, representing different *gp60* subtypes, collected at rural sites in Gabon, Ghana, Madagascar and Tanzania. Phylogenetic and cluster analyses based on single-nucleotide polymorphisms showed that isolates predominantly clustered by their country of origin, irrespective of their *gp60* subtype. We found a significant isolation-by-distance signature that shows the importance of local transmission, but we also detected evidence of hybridization between isolates of different geographical regions. We identified 37 outlier genes with exceptionally high nucleotide diversity, and this group is significantly enriched for genes encoding extracellular proteins and signal peptides. Furthermore, these genes are found more often than expected in recombinant regions, and they show a distinct signature of positive or balancing selection. We conclude that: (1) the metapopulation structure of *C. hominis* can only be accurately captured by whole-genome analyses; (2) local anthroponotic transmission underpins the spread of this pathogen in Africa; (3) hybridization occurs between distinct geographical lineages; and (4) genetic introgression provides novel substrate for positive or balancing selection in genes involved in host–parasite coevolution.

## Data Summary

All raw and processed sequencing data generated and analysed during the current study have been submitted to the National Center for Biotechnology Information (NCBI) Sequence Read Archive (https://www.ncbi.nlm.nih.gov/bioproject/) under the BioProject PRJNA634705.

**Impact Statement**

The evolution and transmission of emerging infectious diseases (EIDs) is affected by human activities and global environmental change. International travel can bring previously isolated parasites and lineages into contact, which can result in genetic exchange due to hybridization. This introgressed variation may form the novel substrate for natural selection, potentially accelerating the evolution of pathogens. Here, we analyse the population genomics of *Cryptosporidium hominis*, an important human pathogen in Africa. Our analyses show that the population structure of the species in Africa is highly fragmented, which is typical for a metapopulation, and that local anthroponotic transmission underpins the spread of this pathogen in Africa. However, we also find evidence of genetic admixture between distinct geographical lineages, which is consistent with spread of this pathogen by long-distance travel, and hybridization between previously isolated strains. We furthermore show that this admixed genetic variation provides novel substrate for positive or balancing selection in genes involved in host–parasite coevolution. This study adds to the growing body of scientific evidence that environmental changes and human activities can not only contribute to the spread of diseases, but can also fuel the evolution of pathogens responsible for EIDs and re-emerging infectious diseases.

## Introduction

The parasitic apicomplexan *Cryptosporidium* is the most important diarrhoea-causing protist worldwide, and it contributes substantially to the paediatric diarrhoeal disease burden in sub-Saharan Africa [1, 2] and Asia [3]. Infections are associated with increasing childhood mortality, malnutrition, cognitive development and persistent growth retardation [1, 4, 5]. In Africa, an estimated 2.9 million cases of cryptosporidiosis occur annually in children <3 years of age, predominantly driven by anthroponotic transmission within neighbourhood clusters [6, 7].

Previous studies have demonstrated that *Cryptosporidium hominis* is the main human pathogen that causes cryptosporidiosis on the African continent [8]. Subtyping of isolates, based on sequence analysis of the highly polymorphic glycoprotein 60 (*gp60*) gene, provides evidence of the co-occurrence of many subtypes from at least six *C. hominis* families (Ia, Ib, Id, Ie, If and Ih) [9]. This high biodiversity is thought to reflect intensive and stable anthroponotic transmission of this parasite [9]. This observation also generates important questions regarding the role of genetic exchanges between the subtypes through hybridization, and whether human-mediated gene flow across Africa may contribute to the evolution of this pathogen.

Recent comparative genomic analyses have advanced our understanding of the evolution, genetic diversity and population structure of *Cryptosporidium* [10, 11]. One study explored the diversity of *C. hominis* isolates collected from children living in the same village in Bangladesh, and it demonstrated high recombination rates and a lack of clustering of the isolates with respect to their *gp60* subtype [12]. Recombination events are also thought to have played a role in the emergence of subtypes with particularly high virulence, including the *C. hominis* IbA10G2 subtype that predominates in high-income countries [13]. Genetic introgression has furthermore been implicated in the adaptation of specific genetic variants of *Cryptosporidium parvum* to the human host [10, 14]. However, to date, no studies have examined *C. hominis* genomes from Africa, and hence, nothing is known about its evolutionary genomics on the continent that is most affected by the pathogen. In the present study, we have performed whole-genome sequencing and comparative genomic analysis of *C. hominis* isolates collected from 26 infected individuals in Gabon, Ghana, Madagascar and Tanzania, and we examined these data to explore the metapopulation structure and genetic introgression, and the genomic signature of host–parasite co-evolution.

## Methods

### Parasite sampling

Sampling was conducted within the frame of a large epidemiological study in sub-Saharan Africa, as described elsewhere [7]. In brief, stool samples were collected from children <5 years presenting to the Outpatient Department (OPD) of four study hospitals with diarrhoea or a history of diarrhoea. All samples were collected from November 2016 to April 2018, in Ghana, Gabon, Madagascar and Tanzania. The study sites were the Agogo Presbyterian Hospital (APH) in the Ashanti Region of Ghana, the Albert Schweitzer Hospital (ASH) in Lambaréné, Gabon, the Imerintsiatosika Health Post (IHP) in the rural outskirts of Antananarivo, Madagascar, and the Korogwe District Hospital (KDH) in the Tanga Region of Tanzania. In case of a positive *Cryptosporidium* rapid diagnostic test (Certest Biotech, Zaragoza, Spain), stool samples were collected from contacts of the index case (household contacts and neighbouring children <5 years). A second stool sample was preserved in 2.5 % potassium dichromate at 4 °C for Whole-genome sequencing (WGS).

### Detection of *Cryptosporidium* and molecular characterization

Genomic DNA from all stool samples was extracted using the DNeasy PowerSoil kit according to the manufacturer’s instructions (Qiagen, Hilden, Germany). *Cryptosporidium* species were identified using a published nested PCR protocol for the amplification and sequencing of the *18S rRNA* gene [15]. All samples positive for *C. hominis* were further subtyped by sequencing an 850 bp fragment of the *gp60* gene using a published nested PCR protocol [16, 17], and assigned to allelic families and subtypes according to a previously proposed nomenclature [18].

### Oocyst purification and next-generation sequencing experiments

A previously published protocol was used [19]. Briefly, stool samples were washed three–five times to remove potassium dichromate, and then suspended in the smallest volume possible (~5 ml) of water. *Cryptosporidium* oocysts were purified from 2 ml of stool suspension by immunomagnetic separation (IMS), using the Dynabeads anti-*Cryptosporidium* kit (Thermo Fisher), according to the instructions indicated by the manufacturer for water samples. To degrade residual bacterial contaminants, purified oocysts were treated with an equal volume of 0.6 % sodium hypochlorite, washed three times with nuclease-free water and pelleted by centrifugation (1100 ***g*** for 5 min). The pellets were suspended in 100 µl of nuclease-free water. An aliquot of 5 µl was used to estimate the number of oocysts by microscopy, whereas the remaining 95 µl were used for DNA extraction.

Considering that a single *Cryptosporidium* oocyst contains 40 fg of DNA, only samples yielding an estimated 2.5×10^4^ highly purified oocysts were processed for DNA extraction. To maximize yield, purified oocysts were first submitted to five cycles of freezing in liquid nitrogen and thawing at 55 °C. Genomic DNA was extracted with the DNA extraction IQ System kit (Promega), following the manufacturer’s instructions, and eluted in 50 µl of elution buffer. DNA concentration was measured using the Qubit dsDNA HS Assay kit and the Qubit 1.0 fluorometer (Invitrogen, Paisley, UK), according to the manufacturer’s instructions.

To assess the presence of residual bacterial DNA in the genomic extracts, a previously described, single round of PCR targeting the *16S rRNA* gene, was used [20]. PCR products were visualized by agarose gel electrophoresis.

Whole-genome amplification (WGA) was performed using the REPLI-g Midi-Kit (Qiagen), according to the manufacturer’s instructions. Briefly, 5 µl of genomic DNA (corresponding to 1–10 ng of genomic DNA) was mixed with 5 µl of denaturing solution and incubated at room temperature for 3 min. Next, 10 µl of stop solution was added to stabilize denatured DNA fragments. The reaction mixture was completed with 29 µl of buffer and 1 µl of phi29 polymerase, and allowed to proceed for 16 h at 30 °C. The reaction was stopped by heating for 5 min at 63 °C. WGA products were visualized by electrophoresis on a 0.7 % agarose gel, purified and quantified by Qubit as described above.

For WGS experiments, ~1 µg of purified WGA products was used to generate Illumina TruSeq 2×150 bp paired-end libraries (average insert size: 500 bp), which were sequenced on an Illumina HiSeq 4000 platform (Illumina, SanDiego, CA, USA). Library preparation and WGS were performed by a sequencing service (GATC, Germany).

### Sequence processing, alignment, variant detection and filtration

Adapter sequences and low-quality bases with an average quality below 20 in a 4 bp sliding window were trimmed using Trimmomatic v.0.36 [21] with the argument SLIDINGWINDOW from paired end sequence reads of the 28 African isolates. Two of the 28 samples (1 from Madagascar and 1 from Tanzania) were excluded (see below), leaving 26 isolates for subsequent analyses.

Filtered reads were then aligned to the *C. hominis* UdeA01 genome [22] using BWA v.0.7 [23] with default parameters, and filtered with the MarkDuplicates option using Picard tools v.2.20.2 (https://github.com/broadinstitute/picard). The genome of the *C. hominis* isolate UdeA01 downloaded from CryptoDB [24] was used as a reference for read mapping. Each isolate had >95 % coverage to at least a fivefold depth [mean coverage 189× (Quartile1=140, Quartile3=195)], with a mean GC content of 31.35 % (Table 1). After quality control, read mapping and joint genotyping, 3128 high-quality SNPs and 896 INDELS were identified in this dataset.

**Table 1.** List of the *C. hominis* isolates included in the study and sequencing statistics

Sample code	Country	Sampling date (yyyy-mm-dd)	GC (%)	% of reference genome covered at >5×	Coverage (mean)	No. of SNPs	SRA accession
Afr1	Ghana	2016-05-18	33	99.4	142X	2810	SRR11843813
Afr2	Ghana	2016-05-16	31	99.9	570X	2797	SRR11843812
Afr3	Ghana	2016-06-02	32	99.8	352X	2782	SRR11843801
Afr5	Ghana	2016-07-25	32	99.8	354X	2788	SRR11843792
Afr6	Ghana	2016-08-12	31	99.9	446X	2797	SRR11843791
Afr7	Ghana	2016-09-20	31	99.8	135X	2953	SRR11843790
Afr9	Ghana	2016-11-22	31	99.8	135X	2953	SRR11843789
Afr10	Madagascar	2017-02-24	31	99.8	156X	2252	SRR11843788
Afr11	Madagascar	2017-03-20	32	93.6	63X	3012	SRR11843787
Afr12	Madagascar	2017-03-22	32	99.8	180X	3055	SRR11843786
Afr13	Madagascar	2017-03-24	31	99.8	182X	3058	SRR11843811
Afr14	Tanzania	2016-12-08	31	99.8	160X	3122	SRR11843810
Afr15	Tanzania	2017-01-02	31	99.8	166X	3010	SRR11843809
Afr16	Tanzania	2017-01-03	31	99.8	154X	2976	SRR11843808
Afr17	Tanzania	2017-01-06	31	99.8	168X	2839	SRR11843807
Afr18	Tanzania	2017-01-30	31	99.8	195X	3015	SRR11843806
Afr20	Tanzania	2017-03-06	32	99.7	143X	3102	SRR11843805
Afr21	Tanzania	2017-03-08	31	99.9	202X	3018	SRR11843804
Afr22	Tanzania	2017-03-13	31	99.8	132X	2831	SRR11843803
Afr23	Tanzania	2017-03-27	31	99.8	173X	2912	SRR11843802
Afr24	Tanzania	2017-03-29	31	99.8	168X	3024	SRR11843800
Afr25	Tanzania	2017-03-29	31	99.8	195X	2826	SRR11843799
Afr26	Tanzania	2017-01-18	31	99.8	156X	2902	SRR11843798
Afr29	Gabon	2017-08-23	32	95.4	18X	2987	SRR11843797
Afr30	Gabon	2017-09-28	32	99.7	162X	3035	SRR11843796
Afr33	Gabon	2017-07-28	32	98.2	40X	3145	SRR11843795
Afr34	Gabon	2018-02-22	31	99.8	202X	3177	SRR11843794
Afr35	Gabon	2017-08-24	31	99.8	131X	3187	SRR11843793

*Cryptosporidium* is an obligate sexual organism, and a single host can be infected by multiple, genetically different parasite populations, which can complicate downstream analyses. For that reason, we employed estMOI [25] software to identify and remove samples representing multiple infections. We used the single most common variant at each nucleotide position of each sample in our population genetic analyses. estMOI software is designed to estimate multiplicity of infection (m.o.i.) based on combinations of polymorphisms from reads in deep sequence data. We sequenced 28 isolates in total, of which 1 (Madagascar isolate Afr10) belonged to the IbA10G2 subtype, which is very uncommon in Africa. This sample also had a lower number of SNPs compared to the other isolates (Fig. S11), and to avoid bias, this isolate was removed from the study. In addition, the Gabon isolate Afr29 was found to represent two infections, as indicated by estMOI’s default parameters. This sample was also removed from the study. In each of the remaining 26 isolates, we identified SNPs and INDELs by using Genome Analysis Toolkit’s (GATK v3.7.0) HaplotypeCaller module [26] with a ploidy 1. The number of high-quality SNPs with this filter, along with the default hard filtering options (Methods in Text S1), as suggested by GATK’s best practices [27], are reported in Table 1. All individual isolates’ VCF files were then jointly genotyped to obtain a combined VCF file and 3128 high-quality SNPs and 896 INDELS were identified in the *C. hominis* African metapopulation. Altogether, these data suggest that the variants called were derived from single infections (except for Afr29, which was excluded).

### PCA, phylogenetic and network analyses

High-quality SNPs were used to visualize clustering among the African isolates using principal component analysis (PCA) implemented in the R package SNPRelate v.1.18 [28]. A Bayesian Markov chain Monte Carlo (MCMC)-based phylogenetic tree was generated using MrBayes v.3.2.6 [29] with the same set of SNPs and visualized in FigTree v.1.4. Additional phylogenies, i.e. maximum-likelihood (ML) and neighbour-joining (NJ)-based trees were generated by using IQTREE [30] and mega7 [31], respectively. A consensus tree was generated using DensiTree 2 [32]. Additionally, a splits network was generated by using neighbour-net algorithm implemented in SplitsTree5 [33].

### Population structure and recombination analyses

Population structure analysis of the isolates was performed with the structure v.2.3 program [34]. The best K value, where K represents the number of subpopulations assumed in structure, was computed using clumpak [35]. Recombination Detection Program v.4 (RDP4) [36] was used to identify potential recombination events, along with breakpoints and major and minor parent contributors. The above set of SNPs were also used to generate consensus sequences of each chromosome for each isolate by using bcftools v1.9 with an argument ‘consensus’ for recombination analysis. Recombination was assessed in RDP4 using the RDP [37], geneconv [38], BootScan [39], MaxChi [40] and Chimaera [41], which were run on multiple sequence alignments of individual chromosomes, with default parameters. Only recombination signals with probability values *P*≤10^−5^ in at least three of the above methods were considered to be significant. Introgression events were visualized using the software HybridCheck [42]. This software was also used to estimate the time of recombinant events, assuming a mutation rate of 10^−8^ per generation. The HybridCheck algorithm dates the events in terms of generation numbers, which were transformed into years [42]. Note that the estimate is conservative (i.e. that the actual date of the introgression may be much earlier) [43]. This is because the minor parent identified in this study may not be the actual parental sequence that has contributed to the genetic exchange, but rather a sequence that is closely related to the identified parental sequence. In this study we refer to the genetic exchanges as admixture rather than as introgression. The latter terminology was used by Nader and colleagues previously [10] when studying the genetic exchanges between *Cryptosporidium parvum parvum* and *Cryptosporidium parvum anthroponosum*. Both genetic introgression and admixture are the consequence of gene flow and recombination. However, with introgression, the lineages exchanging sequence variation have been reproductively isolated for a considerable time, accruing significant genetic divergence in the process. Genetic exchanges at the level of metapopulations are more correctly referred to as admixture, although there is no clear separation between both processes, and both form part of a continuum.

### Population genetic analyses

SNP and INDEL density was calculated using VCFtools, with 1 kb bins [44]. To estimate linkage disequilibrium, the squared correlation coefficient (*r* ^2^) was calculated for each SNP pair within 30 kb by using plink v.1.9 [45]. Nucleotide diversity (π), Tajima’s *D*, Fst and haplotype diversity (*Hd*) were calculated in the African *C. hominis* parasite metapopulation for each polymorphic gene using DnaSP v5 [46]. McDonald and Kreitman’s test (MK) and Hudson, Kreitman and Aguadé’s test (HKA) were also performed in DNASP v.5 [46]. Outlier genes with the highest nucleotide diversity were defined as genes that were >4.0 StDev away from the mean of the distribution. Outliers were removed, sequentially. Nonsynonymous and synonymous substitution rates (denoted as *K*_a_ and *K*_s_, respectively) were estimated using the software KaKs_Calculator [47], with the Nei and Gojobori method. Extra-cellular localization of protein was predicted with WoLF PSORT [48], whereas predicted protein targeting (signalling peptides) genes were obtained from CryptoDB [24]. All the results were plotted with the R statistical programming language (version 3.6.1).

## Results

### Phylogenetic analyses

**Fig. 1.**
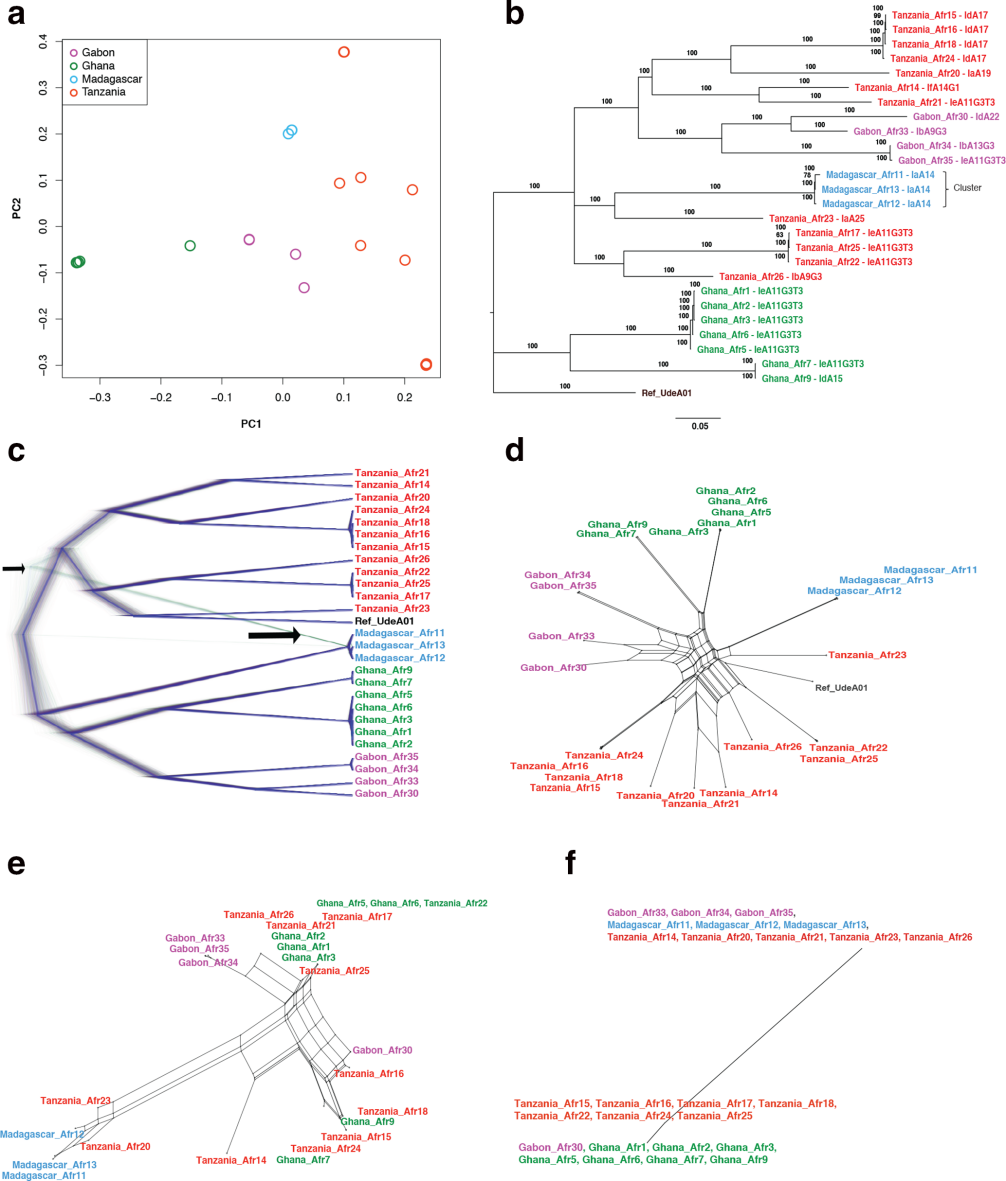
Phylogenetic relationships among *C. hominis* African isolates. Isolates are coloured according to their geographical origin in panels (a–f). (a) Principal component analysis (PCA), where PC1 and PC2 account for variability among *C. hominis* African isolates, differentiating them by physical boundaries. (b) Phylogenetic analysis inferred by Bayesian inference (MrBayes) using a concatenated set of 3128 genomic SNPs. (c) A cloudogram of 2130000 trees obtained by DensiTree. The consensus tree is represented in dark blue. Note the transparent green branches (indicated by the arrow), which reveals that a small part of the Madagascar genomes has a different phylogenetic origin. (d) A phylogenetic network based on all genomic SNPs. The network shows geographical clustering of African isolates by the country of origin. The loops in the network are suggestive of recombination events both within and between isolates from different countries. (e) Network of the *gp60* gene reveals admixture of samples from Madagascar and Tanzania. (f) Network of the CHUDEA6_5260 gene shows no evidence of admixture.

A PCA based on 3128 high-quality SNPs demonstrated that the 26 isolates (Table 1) broadly clustered with respect to their geographical origin (Fig. 1a).

The first principal component (PC1) explains ~22 % of the total variation, and it separates West African (Ghana) from East African (Tanzania) isolates. PC2 separates Central Africa (Gabon) from East Africa (Madagascar), and explains ~18 % of the variation (Fig. 1a). Furthermore, PC3 explains 15 % of the variation, but it does not separate samples geographically and we were unable to identify any driver of this clustering.

A phylogenetic analysis was conducted by Bayesian inference (MrBayes) using a concatenated set of genomic SNPs. The phylogenetic tree was concordant with the PCA analysis and revealed clear geographical segregation (Fig. 1b). Indeed, the majority of isolates from the same country are grouped together under the same clade. The Tanzanian isolates (*n*=12) clustered in two clades; one as a sister clade of the Madagascar isolates and the other as a sister clade of the Gabon isolates. A maximum-likelihood tree (Fig. S1a, available in the online version of this article) mirrors Fig. 1b, but surprisingly, a neighbour-joining tree shows Tanzanian isolates as a completely independent clade (Fig. S1b).

In order to examine this discrepancy in tree topology in further detail, we constructed a consensus tree using DensiTree 2 software. This showed four separate population clusters, and the dark blue branches indicate that these relationships are well supported by a nearly consistent phylogenetic signal (Fig. 1c). Interestingly, the Madagascar samples show transparent green branches (indicated by the arrow), which indicates that a small part of these *C. hominis* genomes has a different phylogenetic origin. The phylogenetic network (Fig. 1d) based on genomic SNPs also shows that the evolutionary origin is not as straightforward as suggested by the main tree topology. The loops in this network are suggestive of recombination events both within and between isolates found in different countries. A network analysis on a gene-by-gene basis revealed the cause of this phylogenetic inconsistency; the topology of the network of virtually all genes shows a clear dichotomy (or trichotomy) between samples from different geographical origins, as illustrated by Fig. 1f (see also Fig. S2). However, the networks of two genes (CHUDEA6_1070 and CHUDEA6_1080, also known as *gp60*) reveal a complex admixture of samples from different geographical origins (Figs 1e and S2). Fig. 1e shows that the samples from Madagascar and Tanzania cluster together, which is consistent with genetic admixture. Note that after removing both genes (CHUDEA6_1070 and CHUDEA6_1080) from the genome dataset, the network still shows loops (data not shown), which implies that there may be other recombination events besides those identified in these two genes.

### Population structure and recombination analyses

**Fig. 2.**
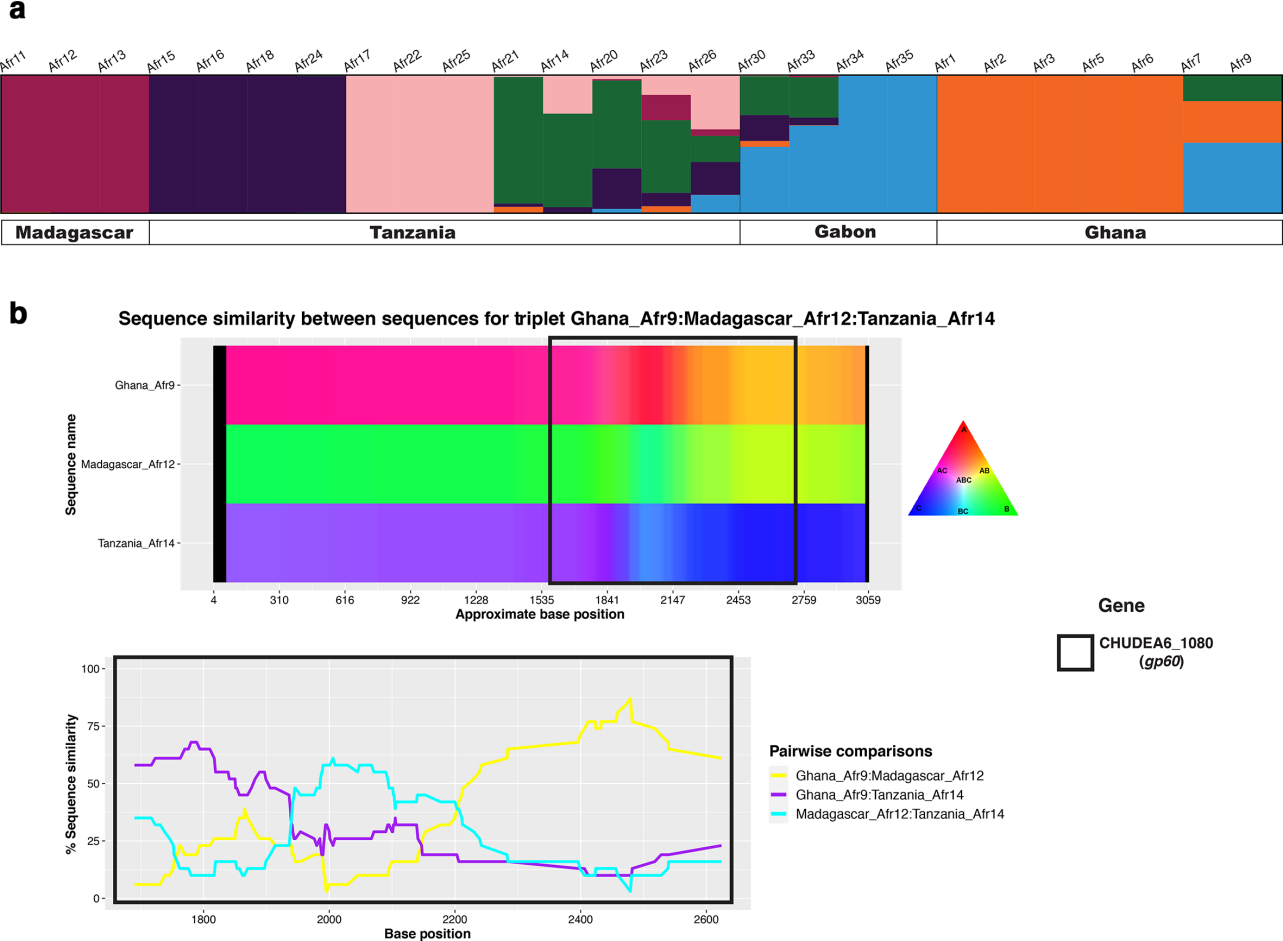
Population structure and recombination analyses. (a) structure plot representing the percentage of shared ancestry among the four African *C. hominis* metapopulation (for K=6). (b) HybridCheck plot illustrating the sequence similarity among the three isolates (Madagascar Afr12, Ghana Afr9 and Tanzania Afr14) involved in recombination at the *gp60* locus.

To analyse population admixture, we studied the proportion of shared ancestry among the African *C. hominis* metapopulation using a structure analysis. Fig. 2a shows the structure graph for K=6, which was identified as the best fitting model for the number of subpopulations (the structure graphs for K=2 to K=10 are illustrated in Fig. S3). Fig. 2a shows that high levels of admixture are observed in isolates from Tanzania and Gabon. The observed admixture is consistent with gene flow between African countries followed by recombination, resulting in several admixed genotypes.

The occurrence of recombination events in the 26 African *C. hominis* isolates was explored further using the RDP4 software. Nine recombination events were detected, all localized to chromosome 6, which contains the *gp60* gene locus (Table S1). Because of the high sequence similarity between some of our samples, we selected four distinct isolates to represent the four African countries (Ghana Afr9, Madagascar Afr12, Tanzania Afr14 and Gabon Afr34). These four sequences were also repeatedly observed either as a recombinant, minor or major parent in the overall analysis (Table S1). Using this reduced dataset, 6 recombination events were identified; all of which were among those observed when all 26 genomes were used for analysis (Table S2).

To further investigate these events, we performed pairwise comparison of SNPs between the four sequences at the recombinant block and at its 5′- and 3′-ends. In particular, we aimed to identify the ‘minor parent’, i.e. the sequence that contributes the insert, and which is introgressed into the ‘major parent’. The pair showing the smallest number of SNP differences at the recombinant block was used to determine the most likely minor parent of the recombinant. The most likely major parent was defined as the sequence with the smallest number of SNP differences at both 5′- and 3′-ends of the recombinant block. Using this approach, we identified the parental sequences of two recombination events (i.e. events 1 and 4, Table S2). In both these events, Madagascar Afr12 was identified as the recombinant, with Ghana Afr9 and Tanzania Afr14 as the minor and major parent, respectively (Tables S3 and S4). Interestingly, event 4 overlapped part of the *gp60* coding region, which corroborates our findings reported in the network analysis (Fig. 1e). Event 3 also overlapped partly with the *gp60* locus, but we failed to identify the parental sequences for this event. We also examined other combinations of the four isolates, which gave consistent results, i.e. evidence of recombination, but in these cases, we were unable to identify the parental sequences. Therefore, in the following analysis we focused on the aforementioned four isolates.

The signature of genetic introgression is illustrated by a graph made with HybridCheck software (Fig. 2b). This figure supports that Madagascar Afr12 is a recombinant that evolved from introgression of Ghana Afr9 (minor parent) into a sequence that resembled Tanzania Afr14 (the major parent). The figure indicates that this recombination event exchanged the nucleotide sequence at the *gp60* gene. Assuming a mutation rate of 10^−8^ per generation, we estimated the age of the recombination event in *gp60* locus based on the nucleotide divergence between the recombinant and the most likely donor. The estimated median age was 476 561 generations with 95 % CI (177 137 – 998 453), which ranged between 2382 (886 – 4992) and 4765 (1771– 9984) years ago for a 48 and 96 h life cycle, respectively. Note that this is a conservative estimate and that the actual recombination event might have been more recent still, because we may not have sampled, sequenced and identified the actual minor parent in this analysis.

To further understand the impact of gene flow and genetic introgression on the population structure of *C. hominis* in Africa, we conducted an isolation-by-distance analysis. In total, 58 % of the genetic diversification was explained by the geographical distance between sampling locations, and interestingly, the relationship appears to be non-linear (quadratic regression: *F*_2,63_ = 46.77, *r* ^2^=0.58, linear component: *P*=1.551e-10, quadratic component: *P*=3.543e-13) (Fig. S4). This shows that the population structure of *C. hominis* in Africa is still largely governed by local transmission, yet that long-distance migration by international travel and genetic introgression have a homogenizing population genetic effect. This could explain the significant quadratic component and the levelling off of the level of genetic differentiation between the most distant geographical locations.

### Polymorphism and analysis of linkage disequilibrium

**Fig. 3.**
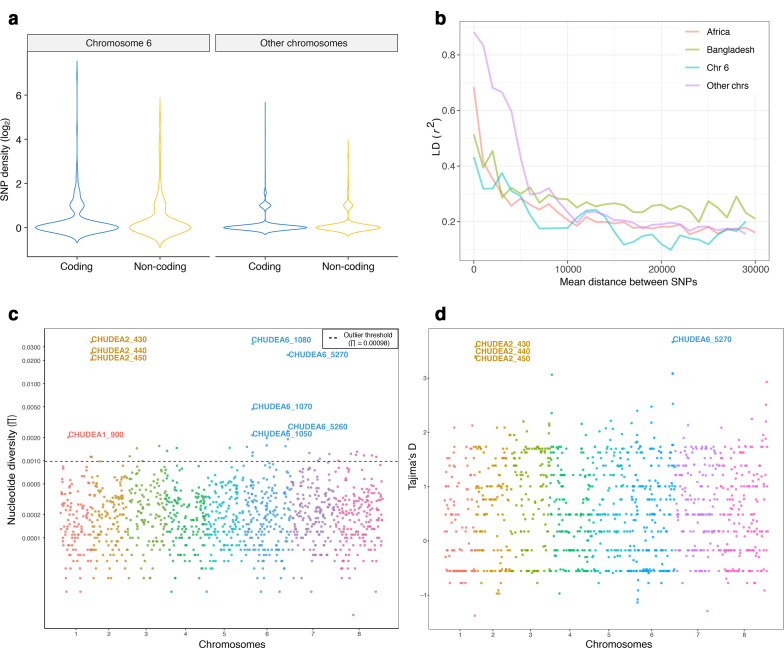
Polymorphism and linkage analyses. Pa(a) Nucleotide variation at chromosome 6 is significantly higher than that of all other chromosomes (GLM: *F*_1,32_=25.16, *P*=5.3e-7), and coding regions show a higher diversity than con-coding regions (GLM: *F*_1,72_=56.65, *P*=5.4e-14). (b) LD decay versus the mean distance between SNPs calculated across the genome for the African metapopulation and the Bangladeshi population. Also shown is the LD decay for chromosome 6 and all other chromosomes in the African metapopulation. The LD decays more rapidly in the African sample compared to the Bangladeshi, and the LD decay is particularly rapid for SNPs in chromosome 6. (c) Nucleotide diversity (*π*) of each polymorphic gene. The 37 genes above the threshold (dashed line) are considered to be outliers, and genes with *π*>0.002 are labelled. (d) Tajima’s *D* values for each polymorphic gene. Genes with >3 Tajima’s *D* values are labelled. In (c) and (d) each chromosome is represented with a different colour.

The density of SNPs and INDELs was compared across the genome (Fig. S5a) and in coding regions only (Fig. S5b). There is a significant difference in nucleotide variation between chromosomes (Kruskal–Wallis test, d.f.=7, *P*=0.021), with chromosome 6 being more polymorphic than all other chromosomes (Wilcox test, *P*=0.0013) (Fig. 3a). Next, a linkage disequilibrium (LD) analysis was performed to estimate recombination frequency within chromosomes in the 26 African *C. hominis* genomes. For comparison, the same analysis was conducted using SNP data from 32 published *C. hominis* genomes from Bangladesh [12]. Fig. 3b shows the decay of LD between SNPs as a function of their genomic distance in the two datasets. In the African metapopulation, the squared correlation coefficient (*r* ^2^) between SNPs decays more rapidly across the African samples, which is consistent with gene flow and recombination between distinct phylogenetic lineages. This would break down haplotypes into smaller fragments, thus reducing linkage disequilibrium. Chromosome 6 has a particularly strong effect on the rapid LD decay across the African metapopulation (Fig. 3b). The analysis of LD thus further supports admixture of samples, as evidenced by our phylogenetic and network analyses (Fig. 1b, d and e), the admixture of samples in the structure analysis (Fig. 2a), and the recombination analysis with RDP4 (Table S5) and HybridCheck (Fig. 2b).

### Identification of highly polymorphic genes

Nucleotide diversity (π) was calculated for each gene across the genome (Fig. 3c). We ranked all genes according to π (from high to low) and examined the function and the location of the protein encoded by each gene. In particular, we determined whether the gene encoded for a protein containing a signal peptide or an extracellular protein. After converting *P*-values into *Q*-values, we found that the first 890 most diverse genes are significantly enriched for signal peptides, and the 236 most diverse genes for extracellular proteins encoding genes (Tables S6 and S7).

A total of 37 genes showed a nucleotide diversity that was >4 standard deviations away from the mean. These 37 outlier genes were significantly enriched for genes encoding for extracellular proteins (binomial probability: *P*=9.38 x 10^−4^) (Figs S6 and S7, Table S8). Most of the outlier genes (34 out of 37, 92 %) have a positive Tajima’s *D* [mean (±se) Tajima’s *D*=1.46 (±0.18)], and this distribution has a mean significantly larger than zero (one-sample *t*-test: d.f.=36, *P*<0.001). The mean Tajima’s *D* value averaged across all other polymorphic genes in the genome [*n*=1142, mean (±se) Tajima’s *D*=0.37 (±0.02)] also significantly exceeds zero (one-sample *t*-test: d.f.=1141, *P*<0.001) (Fig. 3d). This indicates a lack of rare nucleotide polymorphisms in the African *C. hominis* genomes analysed here. Such population genetic signature is unlikely to be the result of positive selection. Rather, the positive Tajima’s *D* is consistent with a recent population expansion after one or more founder events from genetically diverged sources. Interestingly, the Tajima’s *D* of the 37 outlier genes was significantly higher than that of the other 1142 polymorphic genes (*t*-test: *P*<0.001). This implies that the 37 outlier genes might be affected by balancing selection, which elevates the haplotype diversity and Tajima’s *D* value at those 37 genes. A (non-exclusive) alternative explanation is that recombination may have elevated the haplotype diversity at these 37 genes. The exchange of nucleotide variation across different haplotypes could also have increased the Tajima’s *D* statistic relative to that of other genes.

There are two hypotheses that can both explain the elevated Tajima’s *D* statistic of the 37 outlier genes; i.e. balancing selection or recombination. In order to test both hypotheses, we examined whether we could detect evidence for selection across the genome. Consistent with our hypothesis, the 37 outlier genes identified above appear to have a significantly higher ratio of non-synonymous to synonymous substitutions [mean (±se) *K*_a_/*K*_s_=0.44 (±0.057), median=0.40, Q1=0.26, Q3=0.57] when compared to the other 1142 polymorphic genes [mean (±se) *K*_a_/*K*_s_=0.31 (±0.014), median=0.27, Q1=0.25, Q3=0.31] (Mann–Whitney test: *P*=0.032). The relatively elevated *K*_a_/*K*_s_ ratio suggests that positive or balancing selection may be operating on the nucleotide variation of these 37 outlier genes. Interestingly, 7 out of the 37 outlier genes (19 %) are located inside recombinant regions, which is significantly more than the 73 out of 1069 (6.4 %) other polymorphic genes (Fisher exact test: *P*=0.0099). This shows that the highly polymorphic outlier genes are found more often in recombinant regions than expected by chance. In other words, we find evidence to support both our hypotheses, and it appears that both recombination and balancing selection contributed to the high level of variation at the 37 outlier genes.

### Analysis of highly variable virulence genes

**Fig. 4.**
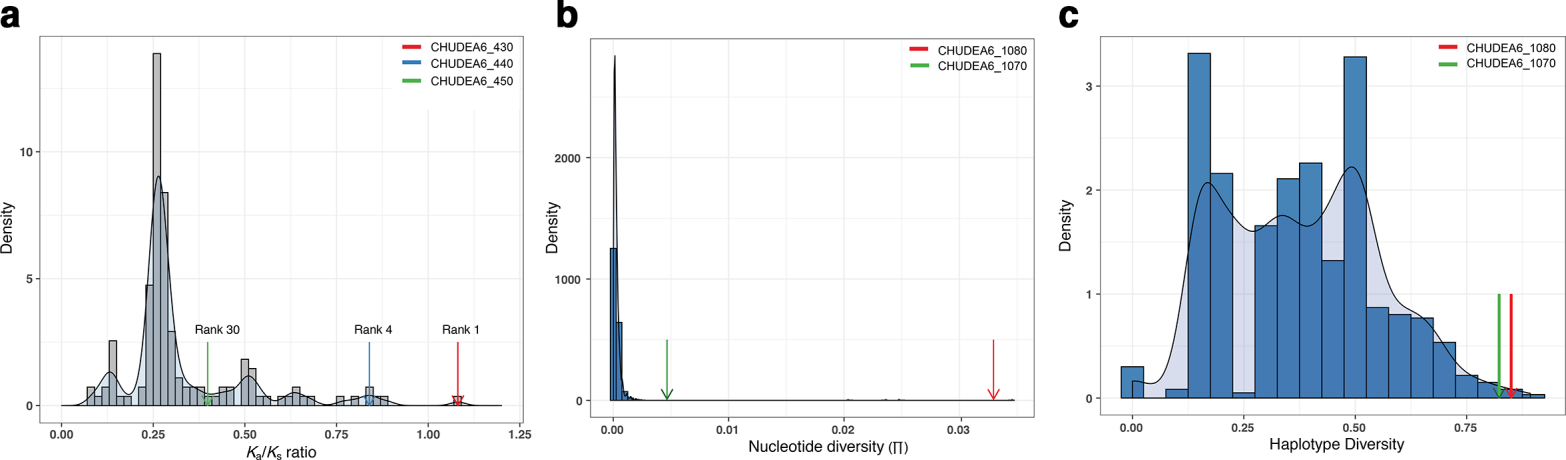
Distribution and density plots. (a) Density of *K*_a_/*K*_s_ ratio of polymorphic genes. *K*_a_/*K*_s_ ratios of the CHUDEA2_430 (red arrow), CHUDEA2_440 (blue arrow) and CHUDEA2_450 (green arrow) genes are labelled along with their ranks. The CHUDEA2_430 gene has the highest *K*_a_/*K*_s_ ratio (*K*_a_/*K*_s_ >1) and hence acts as an outlier. Panels (b) and (c) represent density plots of polymorphic genes in recombinant and recombinant-free regions. (b) Nucleotide diversity. (c) Haplotype diversity. Green and red arrows indicate values for the CHUDEA6_1070 and CHUDEA6_1080 (*gp60*) genes, respectively, in each panel.

Finally, we focus on two genetic loci with extraordinary levels of nucleotide diversity, conducting more detailed population genetic analyses to understand the evolutionary forces that may have contributed to this variation. Firstly, three genes on chromosome 2 (CHUDEA2_430, CHUDEA2_440 and CHUDEA2_450) show extremely high nucleotide diversity. The orthologues of these genes in another closely associated human infective parasite, *C. parvum*, are members of a well-known mucin-like gene family consisting of seven gene members in total [49]. The highly polymorphic three genes identified here all encode for extracellular proteins, whereas two present a signal peptide, and one possesses a transmembrane domain (Table S9). The SNPs across these genes are in complete linkage disequilibrium (LD=1; Fig. S8), which suggests they either represent a co-adapted gene complex, and/or that they have been introgressed as a single block into the *C. hominis* genome. The analyses with RDP4 and HybridCheck did not highlight this as a recombinant genomic region. Nevertheless, the two haplotypes are highly diverged: a total of 84 SNPs in 1560 nucleotides (Figs S9 and S10), and one of the two haplotypes is only observed in Gabon and Tanzania (Table S10). Furthermore, the nucleotide variation of the other four mucin-like member genes of this gene family [mean (±sd)π=0.000275 (±0.00055)] is not higher than that of the chromosome 2 average [mean (±sd)π=0.0009 (±0.0040)]. This suggests that the variation at the three highly polymorphic genes in chromosome 2 might be from heterospecific origin and only recently introgressed into the *C. hominis* genome. Interestingly, Gabon is fixed for the alternative haplotype (i.e. the ‘Gabon type’), whereas Tanzania has both haplotypes in nearly equal frequencies (Afr 14, 15, 16, 18, 20, 21 and 24 are the ‘Gabon type’, and Afr 17, 22, 23, 25, 26 are the ‘common type’), whilst in the other locations in Africa we only observed the common type (Figs S9 and S10). The considerable haplotype diversity in Tanzania suggests that this polymorphism might be under balancing selection. Furthermore, all three genes show a Tajima’s *D* >3.3 (Fig. 3d, Table S9), and one of these genes (CHUDEA2_430) has a *K*_a_/*K*_s_>1, which is the highest ratio of nonsynonymous to synonymous substitutions (Fig. 4a). These data suggest that the variation at these genes is not evolving neutrally, and that it might be under balancing selection.

The second noteworthy observation is the large number of highly variable genes located on chromosome 6 (Fig. 3c). Chromosome 6 is the most polymorphic of the chromosomes, and it shows the most rapid decay of LD, which highlights the prominent role of recombination. Two genes on this chromosome, i.e. CHUDEA6_1070 and CHUDEA6_1080 (*gp60*), fall in the tail of the density distributions of the nucleotide diversity (π) (Fig. 4b) and haplotype diversity (*H*) (Fig. 4c), and both are significant outliers (Wilcoxon rank sum tests: nucleotide diversity, *P*=0.015; haplotype diversity, *P*=0.014). We conducted a neutrality test (McDonald–Kreitman test) on CHUDEA6_1070 and CHUDEA6_1080, comparing the synonymous and nonsynonymous variation within *C. hominis* African metapopulation, and comparing this relative to the divergence with *C. parvum*. The McDonald–Kreitman test was significant for CHUDEA6_1080 (Fisher’s exact test: *P*=0.007) but non-significant for CHUDEA6_1070 (Fisher’s exact test: *P*=1.00). This suggests that CHUDEA6_1080 (*gp60*) is under positive selection, but that the elevated level of nucleotide variation in the adjacent gene CHUDEA6_1070 is likely the result of recombination and genetic hitchhiking alongside *gp60*.

## Discussion

Cryptosporidiosis is a leading cause of mortality, malnutrition and cognitive deficits in African children, yet much of the biology of the pathogens that cause this disease remains unknown. Here we present the first evolutionary genomics analysis of *Cryptosporidium hominis* from Africa, the main pathogen that causes cryptosporidiosis on this continent. We show how gene flow among African countries and recombination between different lineages have generated novel haplotypes. Furthermore, this sequence exchange resulted in significant population admixture between lineages from different geographical origins. In our genome-wide analysis, we identified a set of 37 outlier genes with elevated level of nucleotide diversity and Tajima’s *D*, and these genes were significantly enriched for genes encoding extracellular proteins and signal peptides. Interestingly, these genes were also found significantly more often in recombinant regions of the genome, and we detected a signature of positive selection based on the pattern of nucleotide substitutions (*K*_a_/*K*_s_) at these genes. We also conducted a more in-depth population genetic analysis on two genetic loci at chromosome 2 and 6, revealing the signature of genetic admixture, positive or balancing selection, and genetic hitchhiking. Next, we will discuss our findings in the light of the population genomics of emerging infectious diseases (EIDs).

Previous studies have focused on the subtyping of isolates using *gp60*, which has revealed the co-occurrence of many subtypes and at least six *C. hominis* families (Ia, Ib, Id, Ie, If and Ih) in Africa [9]. Given the increase in international travel, human-mediated gene flow may facilitate genetic exchanges between *C. hominis* subtypes and families through hybridization, thereby potentially contributing to the evolution of this pathogen. Indeed, the evolution and population genomic structure of *Cryptosporidium* parasites is relatively complex due to the interaction of gene flow and recombination, resulting in genetic introgression [10, 50]. In the case of *C. parvum* (zoonotic), strong geographical structuring has been observed based on *gp60* subtyping, consistent with isolation-by-distance and low levels of gene flow [10]. On the other hand, the population structure of *C. hominis*, at least in Europe and other high-income countries, is thought to be strongly influenced by global travel (imported disease), which erodes the isolation-by-distance signal [10].

In this study, we conducted a comparative genomic analysis of 26 *C*. *hominis* isolates collected during a large epidemiological study of transmission dynamics of *Cryptosporidium* in Gabon, Ghana, Madagascar and Tanzania [6, 7]. Our examinations revealed clear geographical clustering of *C. hominis* isolates at the country level and a significant isolation-by-distance signature. The non-linear relationship between geographicl distance and genetic diversification (*Dxy*) suggests that the population structure of *C. hominis* is largely influenced by local movement, and that long-distance migration by international travel and genetic admixture limits the continent-scale genomic diversification.

Our analyses indicate that the clusters observed using whole-genome SNPs do not reflect the *gp60* subtype, and they highlight the importance of (long-distance) gene flow and recombination for *C. hominis* in the African metapopulation. We show evidence of genetic admixture between lineages, and in particular that some of the genomes observed in Madagascar are recombinants that have evolved from genetic contribution of a Ghanaian into a Tanzanian ancestral sequence. The exchange involved the nucleotide sequences at the *gp60* gene, and we estimate this occurred between 886–4992 and 1771–9984 years ago, assuming a 48 and 96 h life cycle, respectively. Our analysis may, however, overestimate the date of genetic admixture (i.e. genetic exchange may have been more recent than estimated here). We believe this is likely because we may not have sampled the actual parental sequences that created the recombinant, making the genetic exchange look older than it actually is. Furthermore, we observed that the signature of genetic admixture was not distributed randomly throughout the genome, but it appeared to be localized in chromosome 6 (and possibly chromosome 2). The importance of recombination was also corroborated by our analysis of the decay in linkage disequilibrium among SNPs, which dropped more rapidly in the African metapopulation than in the Bangladeshi *C. hominis* population [12], and the rate of LD decay was most rapid for chromosome 6.

Next, we will discuss the potential role of genetic admixture in the adaptive evolution of *C. hominis* in the African metapopulation. We only detected a small number of recombination events that were significantly supported by different algorithms, and these were located on chromosome 6. Recombination in a small region of this chromosome that includes *gp60* was associated with significantly elevated haplotype and nucleotide diversity. A gene adjacent to *gp60* also showed an elevated level of nucleotide variation and it was located in the same recombination block as *gp60*. However, this gene did not show the hallmarks of ancient positive selection according to the McDonald–Kreitman test. We therefore conclude that its high nucleotide variation is not the consequence of adaptive evolution, but rather the result of recombination and genetic hitchhiking alongside *gp60*.

In addition, 3 genes in chromosome 2 (CHUDEA2_430, 440 and 450) possessed 2 main haplotypes with an extremely high nucleotide divergence, and these 84 SNPs were in complete linkage disequilibrium. These three genes are members of a well-known mucin-like gene family [49], and based on the high haplotype divergence and LD, we believe that this polymorphism is a consequence of recent genetic introgression from a diverged subtype or (sub)species of *Cryptosporidium*. These three genes also display extensive polymorphisms between *C. parvum* and *C. hominis* (71–79 % identity) [49], which suggests that they are targets for diversifying selection. An *in vivo* study implicated these genes in the process of host cell invasion, which makes them important virulence determinants adapted to particular host species [49]. These genes have also been suggested as vaccine targets [51, 52]. However, the significant standing variation present at these genes implies that this pathogen would be able to rapidly evolve polymorphisms at these genes, thus escaping vaccine recognition.

We identified 37 highly polymorphic genes throughout the genome that displayed an excessive level of diversity (i.e. elevated Tajima’s *D*), and many of these genes encode extracellular proteins and signal peptides. A positive Tajima’s *D* value indicates that there are more distinct haplotypes than expected based on the number of segregating sites present at these genes. Besides demographic effects and balancing selection, this can also be caused by recombination, when the same nucleotide polymorphisms are exchanged between different haplotypes. Such an exchange elevates haplotype diversity but not the number of segregating sites at these genes. The Tajima’s *D* of the group of highly polymorphic genes was not only positive, but it was also significantly higher than the genome average. This implies that demographic effects and recombination cannot be solely responsible for the elevated Tajima’s *D* at these genes, given that both these processes are expected to affect all genes to a similar extent. Rather, we propose that the haplotype diversity generated by recombination in the highly polymorphic genes may subsequently have been targeted by positive or balancing selection. We supported this finding by showing a significant elevation in the ratio of non-synonymous to synonymous substitutions (*K*_a_/*K*_s_) of these highly polymorphic genes, and the fact that these genes are found more often than expected in recombinant regions. Adaptive admixture and introgression enable pathogens to rapidly adapt to the immune systems of hosts. For example, influenza viral pathogens of humans are able to evade host immunity and reinfect previously infected individuals through viral reassortment [53]. However, the role of adaptive admixture or introgression in other human pathogens is much less well established (but see [54, 55]). Given the large number of subtypes [9], and the signature of natural selection and recombination in the genome of *C. hominis*, we believe that adaptive introgression could play an important role in the virulence evolution of this pathogen in Africa.

We acknowledge that our analyses are based on a relatively small number of whole-genome sequences, which means that our conclusions need to be treated with caution, and that the inference we make about the adaptive significance of genetic admixture is indirect. Nevertheless, we tentatively propose that genetic admixture of diverged lineages provides novel nucleotide variation across the genome, elevating the genome-wide Tajima’s *D* statistic. Natural selection (balancing or positive selection) operates on the novel nucleotide variation of a small number of genes, including the *gp60* gene, the mucin-like genes on chromosome 2, and potentially other genes that encode extracellular proteins and signal peptides. In turn, this elevates the Tajima’s *D* and *K*_a_/*K*_s_ value, as revealed by our analyses. Based on these results, we propose that some of this recombinant variation may be adaptive, thereby helping to drive the co-evolutionary arms race between *C. hominis* and its human host in Africa.

In conclusion, this work has demonstrated clear, broad geographical structuring of *C. hominis* isolates across four African countries, and it revealed some fascinating evolution. In particular, we show that genetic exchanges occur between geographically diverged lineages of *C. hominis*, and that this signature of recombination is most apparent in genes that encode for cell surface and secreted protein involved in interactions between the parasite’s invasive stages and the host cells. We propose that the observed sequence exchange contributes novel genetic variation that may become a substrate for natural selection, and that this recombinant variation may therefore be fuelling the co-evolutionary arms race of this pathogen against its human host.

## Supplementary Data

Supplementary material 1

## References

[1] Kotloff KL, Nataro JP, Blackwelder WC, Nasrin D, Farag TH (2013). Burden and aetiology of diarrhoeal disease in infants and young children in developing countries (the global enteric multicenter study, GEMs): a prospective, case-control study. Lancet.

[2] Krumkamp R, Sarpong N, Schwarz NG, Adlkofer J, Loag W (2015). Gastrointestinal infections and diarrheal disease in Ghanaian infants and children: an outpatient case-control study. PLoS Negl Trop Dis.

[3] Sow SO, Muhsen K, Nasrin D, Blackwelder WC, Wu Y (2016). The Burden of *Cryptosporidium* diarrheal disease among children. PLoS Negl Trop Dis.

[4] Molbak K, Andersen M, Aaby P, Hojlyng N, Jakobsen M (1997). Cryptosporidium infection in infancy as a cause of malnutrition: a community study from Guinea-Bissau, West Africa. Am J Clin Nutr.

[5] Guerrant DI, Moore SR, Lima AA, Patrick PD, Schorling JB (1999). Association of early childhood diarrhea and cryptosporidiosis with impaired physical fitness and cognitive function four-seven years later in a poor urban community in northeast Brazil. Am J Trop Med Hyg.

[6] Sow SO, Muhsen K, Nasrin D, Blackwelder WC, Wu Y (2016). The burden of *Cryptosporidium* diarrheal disease among Children. PLoS Negl Trop Dis.

[7] Krumkamp R, Aldrich C, Maiga-Ascofare O, Mbwana J, Rakotozandrindrainy N (2020). Transmission of Cryptosporidium spp. among human and animal local contact networks in sub-Saharan Africa: a multi-country study. Clin Infect Dis.

[8] Aldeyarbi HM, Abu El-Ezz NMT, Karanis P (2016). *Cryptosporidium* and cryptosporidiosis: the African perspective. Environ Sci Pollut Res Int.

[9] Squire SA, Ryan U (2017). *Cryptosporidium* and Giardia in Africa: current and future challenges. Parasites & Vectors.

[10] Nader JL, Mathers TC, Ward BJ, Pachebat JA, Swain MT (2019). Evolutionary genomics of anthroponosis in *Cryptosporidium*. Nat Microbiol.

[11] Fan Y, Feng Y, Xiao L (2019). Comparative genomics: how has it advanced our knowledge of cryptosporidiosis epidemiology?. Parasitol Res.

[12] Gilchrist CA, Cotton JA, Burkey C, Arju T, Gilmartin A (2018). Genetic diversity of *Cryptosporidium hominis* in a Bangladeshi community as revealed by whole-genome sequencing. J Infect Dis.

[13] Guo Y, Tang K, Rowe LA, Li N, Roellig DM (2015). Comparative genomic analysis reveals occurrence of genetic recombination in virulent *Cryptosporidium hominis* subtypes and telomeric gene duplications in *Cryptosporidium parvum*. BMC genomics.

[14] Xiao L (2010). Molecular epidemiology of cryptosporidiosis: an update. Exp Parasitol.

[15] Xiao L, Morgan UM, Limor J, Escalante A, Arrowood M (1999). Genetic diversity within *Cryptosporidium parvum* and related *Cryptosporidium* species. Applied and environmental microbiology.

[16] Alves M, Xiao L, Sulaiman I, Lal AA, Matos O (2003). Subgenotype analysis of *Cryptosporidium* isolates from humans, cattle, and zoo ruminants in Portugal. J Clin Microbiol.

[17] Strong WB, Gut J, Nelson RG (2000). Cloning and sequence analysis of a highly polymorphic *Cryptosporidium parvum* gene encoding a 60-kilodalton glycoprotein and characterization of its 15- and 45-kilodalton zoite surface antigen products. Infection and immunity.

[18] Sulaiman IM, Hira PR, Zhou L, Al-Ali FM, Al-Shelahi FA (2005). Unique endemicity of cryptosporidiosis in children in Kuwait. J Clin Microbiol.

[19] Hadfield SJ, Pachebat JA, Swain MT, Robinson G, Cameron SJ (2015). Generation of whole genome sequences of new Cryptosporidium hominis and Cryptosporidium parvum isolates directly from stool samples. BMC Genomics.

[20] Kommedal Ø, Simmon K, Karaca D, Langeland N, Wiker HG (2012). Dual priming oligonucleotides for broad-range amplification of the bacterial 16S rRNA gene directly from human clinical specimens. J Clin Microbiol.

[21] Bolger AM, Lohse M, Usadel B (2014). Trimmomatic: a flexible trimmer for illumina sequence data. Bioinformatics.

[22] Isaza JP, Galván AL, Polanco V, Huang B, Matveyev AV (2015). Revisiting the reference genomes of human pathogenic Cryptosporidium species: reannotation of *C. parvum* Iowa and a new *C. hominis* reference. Sci Rep.

[23] Li H, Durbin R (2009). Fast and accurate short read alignment with Burrows-Wheeler transform. Bioinformatics.

[24] Heiges M, Wang H, Robinson E, Aurrecoechea C, Gao X (2006). CryptoDB: a Cryptosporidium bioinformatics resource update. Nucleic Acids Res.

[25] Assefa SA, Preston MD, Campino S, Ocholla H, Sutherland CJ (2014). estMOI: estimating multiplicity of infection using parasite deep sequencing data. Bioinformatics.

[26] McKenna A, Hanna M, Banks E, Sivachenko A, Cibulskis K (2010). The genome analysis toolkit: a MapReduce framework for analyzing next-generation DNA sequencing data. Genome Research.

[27] Van der Auwera GA, Carneiro MO, Hartl C, Poplin R, Del Angel G (2013). From FastQ data to high confidence variant calls: the genome analysis toolkit best practices pipeline. Curr Protoc Bioinformatics.

[28] Zheng X, Levine D, Shen J, Gogarten SM, Laurie C (2012). A high-performance computing toolset for relatedness and principal component analysis of SNP data. Bioinformatics.

[29] Ronquist F, Teslenko M, van der Mark P, Ayres DL, Darling A (2012). MrBayes 3.2: efficient Bayesian phylogenetic inference and model choice across a large model space. Systematic Biology.

[30] Nguyen LT, Schmidt HA, von Haeseler A, Minh BQ (2015). IQ-TREE: a fast and effective stochastic algorithm for estimating maximum-likelihood phylogenies. Mol Bio Evol.

[31] Stecher G, Tamura K, Kumar S (2020). Molecular evolutionary genetics analysis (MEGA) for macOS. Mol Bio Evol.

[32] Bouckaert RR, Heled J (2014). DensiTree 2: seeing trees through the forest. bioRxiv.

[33] Huson DH, Bryant D (2006). Application of phylogenetic networks in evolutionary studies. Mol Bio Evol.

[34] Pritchard JK, Stephens M, Donnelly P (2000). Inference of population structure using multilocus genotype data. Genetics.

[35] Kopelman NM, Mayzel J, Jakobsson M, Rosenberg NA, Mayrose I (2015). Clumpak: a program for identifying clustering modes and packaging population structure inferences across K. Mol Ecol Resour.

[36] Martin DP, Murrell B, Golden M, Khoosal A, Muhire B (2015). RDP4: detection and analysis of recombination patterns in virus genomes. Virus Evolution.

[37] Martin D, Rybicki E (2000). RDP: detection of recombination amongst aligned sequences. Bioinformatics.

[38] Sawyer S (1999). GENECONV: a computer package for the statistical detection of gene conversion. http://www math wustl edu/~ sawyer. http://www%20math%20wustl%20edu/~%20sawyer.

[39] Salminen MO, CARR JK (1995). BURKE DS, McCUTCHAN FE. Identification of breakpoints in intergenotypic recombinants of HIV type 1 by bootscanning. AIDS Research and Human Retroviruses.

[40] Smith JM (1992). Analyzing the mosaic structure of genes. J Mol Evol.

[41] Posada D, Crandall KA (2001). Evaluation of methods for detecting recombination from DNA sequences: computer simulations. Proceedings of the National Academy of Sciences.

[42] Ward BJ, van Oosterhout C (2016). HYBRIDCHECK: software for the rapid detection, visualization and dating of recombinant regions in genome sequence data. Mol Ecol Resour.

[43] Jouet A, McMullan M, van Oosterhout C (2015). The effects of recombination, mutation and selection on the evolution of the Rp1 resistance genes in grasses. Mol Ecol.

[44] Danecek P, Auton A, Abecasis G, Albers CA, Banks E (2011). The variant call format and VCFtools. Bioinformatics.

[45] Purcell S, Neale B, Todd-Brown K, Thomas L, Ferreira MA (2007). PLINK: a tool set for whole-genome association and population-based linkage analyses. Am J Hum Genet.

[46] Librado P, Rozas J (2009). DnaSP V5: a software for comprehensive analysis of DNA polymorphism data. Bioinformatics.

[47] Zhang Z, Li J, Zhao XQ, Wang J, Wong GK (2006). KaKs_Calculator: calculating KA and Ks through model selection and model averaging. Genomics Proteomics Bioinformatics.

[48] Horton P, Park K-J, Obayashi T, Fujita N, Harada H (2007). Wolf PSORT: protein localization predictor. Nucleic acids research.

[49] O'Connor RM, Burns PB, Ha-Ngoc T, Scarpato K, Khan W (2009). Polymorphic mucin antigens CpMuc4 and CpMuc5 are integral to Cryptosporidium parvum infection *in vitro*. Eukaryot Cell.

[50] Feng Y, Ryan UM, Xiao L (2018). Genetic diversity and population structure of Cryptosporidium. Trends Parasitol.

[51] Ifeonu OO, Simon R, Tennant SM, Sheoran AS, Daly MC (2016). Cryptosporidium hominis gene catalog: a resource for the selection of novel Cryptosporidium vaccine candidates. Database.

[52] Checkley W, White AC, Jaganath D, Arrowood MJ, Chalmers RM (2015). A review of the global burden, novel diagnostics, therapeutics, and vaccine targets for Cryptosporidium. Lancet Infect Dis.

[53] Arnold ML, Kunte K (2017). Adaptive genetic exchange: a Tangled history of admixture and evolutionary innovation. Trends Ecol Evol.

[54] Maxwell CS, Mattox K, Turissini DA, Teixeira MM, Barker BM (2019). Gene exchange between two divergent species of the fungal human pathogen, Coccidioides. Evolution.

[55] van Dorp L, Gelabert P, Rieux A, de Manuel M, de-Dios T (2020). Plasmodium vivax malaria viewed through the lens of an Eradicated European strain. Molecular biology and evolution.

