# Supplementary material for: Comparative genomics revealed adaptive admixture in Cryptosporidium hominis in Africa
Source: Microb Genom. 2020 Dec 23;7(1):mgen000493. doi: 10.1099/mgen.0.000493 (PMC8115899; doi:10.1099/mgen.0.000493)
Supplement: Supplementary material 1 [file mgen-7-493-s001.pdf]

Supplemental Methods: SM1

SM1 – Sequence processing, alignment, variant detection and filtration

SNPs and INDELs identified by GATK's best practices [1], were separated and filtered independently SNPs with QualByDepth (QD) < 2.0, FisherStrand (FS) > 60.0, RMSMappingQuality (MQ) < 40.0, MappingQualityRankSumTest (MQRankSum) < -12.5, ReadPosRankSumTest (ReadPosRankSum) < -8.0 and StrandOddsRatio (SOR) > 4.0" were filtered out to eliminate false positives. Likewise, INDELs with QualByDepth (QD) < 2.0, FisherStrand (FS) > 60.0, ReadPosRankSumTest (ReadPosRankSum) < -8.0 and StrandOddsRatio (SOR) > 4.0" were filtered out. Furthermore, SNPs and INDELSs with QUAL < 30, allele depth < 5 were removed. A minor allele frequency (MAF) of 0.05 was considered for subsequent sequence analysis.

Supplemental Information: SI1

SI1 – Biological significance of top seven out of 37 outlier genes

Top seven genes out of 37 outlier genes with a level of nucleotide variation that is higher than the genome-wide average and which are polymorphic on chromosome 2 and 6 (Fig. 4C). This gene set is enriched for genes encoding extracellular proteins and signal peptides. On chromosome 2 (LN877948), the variable genes included CHUDEA2_430, CHUDEA2_440 and CHUDEA2_450, which are located in a sub-telomeric region (Additional file 2: Table S6). Of note, the orthologous genes in the closely related species *C. parvum* are known to encode for mucins containing signal peptides [2]. All of these genes display extensive polymorphisms between and within *C. parvum* and *C. hominis* (56-83% identity), which has led to the hypothesis that the gene products are important virulence determinants subject to immune pressure [2].

On chromosome 6 (LN877952), the most polymorphic genes were the *gp60* gene (CHUDEA6_1080) and the adjacent gene (CHUDEA6_1070), which has unknown function. Both genes showed particularly high nucleotide diversity in a recent comparative genomics study of *C. hominis* isolates from Bangladesh [3]. In the present study, we show that these genes have been subject to genetic admixture, which is likely to have elevated their gene diversity. The *C. parvum* ortholog

(cgd2_5260) of CHUDEA6_5260, which encodes an ABC transporter, was identified among the highly variable genes in a comparative genomics study of *C. parvum* isolates with different host specificity [4], while the CHUDEA6_5270 gene was among the highly polymorphic genes identified in a comparison of *C. hominis* genomes from the USA [5].

Figure S1 – A. Maximum-likelihood phylogenetic tree generated by using IQ-TREE [6], showing that the Tanzanian isolates are comprised in two clades. **B.** Neighbor-joining phylogenetic tree generated with MEGA [7], showing four independent clades that correspond to the four African countries.

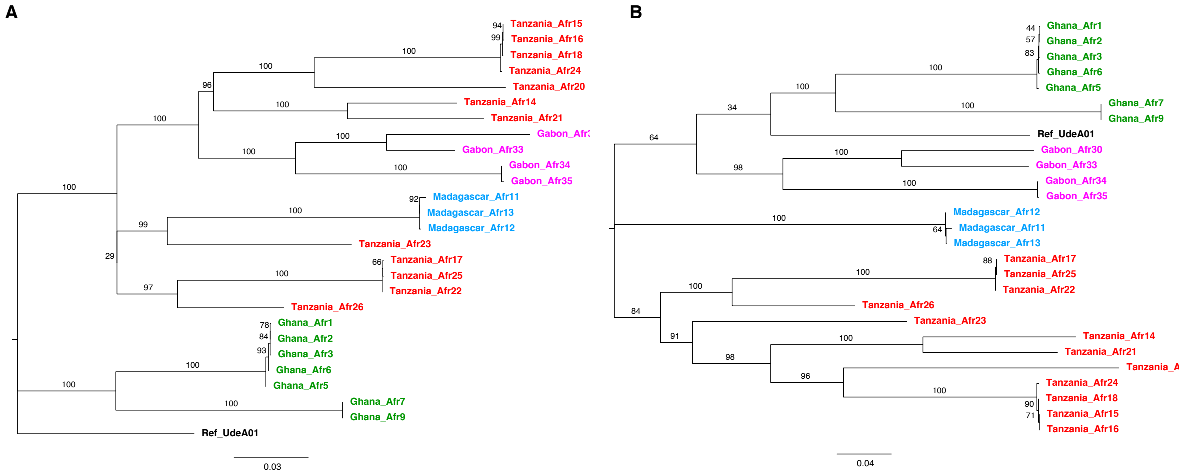

Figure S2 – Network analysis [8] of polymorphic genes. The topology of most genes shows a clear dichotomy (or trichotomy) except for CHUDEA6_1070 and CHUDEA6_1080 (*gp60*). The complex networks of these two genes are consistent with high level of polymorphism, and the loops suggest that these polymorphisms were exchanged between isolates by recombination. A. CHUDEA1_900. B. CHUDEA2_430. C. CHUDEA2_440. D. CHUDEA2_450. E. CHUDEA6_1050. F. CHUDEA6_1070. G. CHUDEA6_1080. H. CHUDEA6_5260. I. CHUDEA6_5270

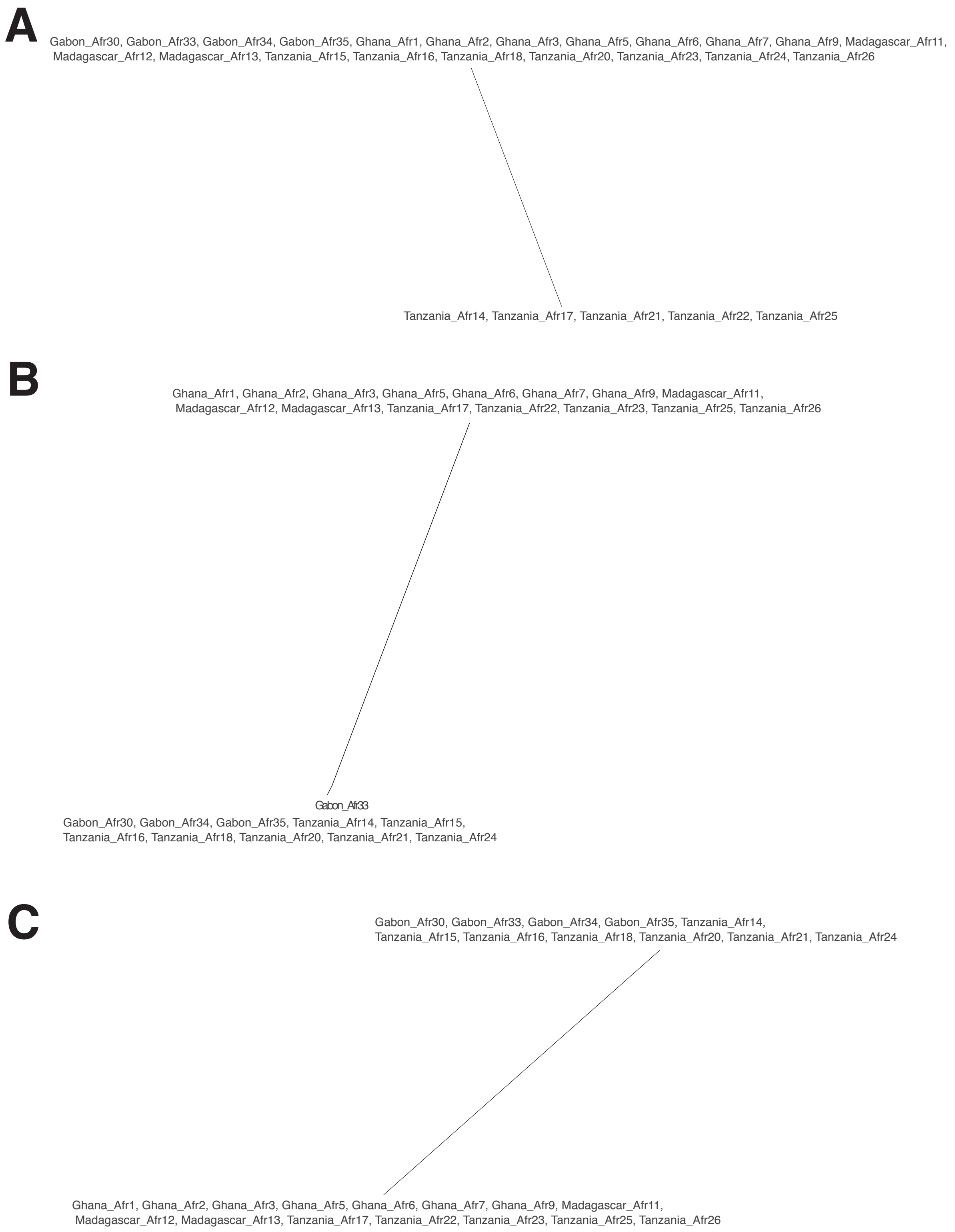
A
Gabon_Afr30, Gabon_Afr33, Gabon_Afr34, Gabon_Afr35, Ghana_Afr1, Ghana_Afr2, Ghana_Afr3, Ghana_Afr5, Ghana_Afr6, Ghana_Afr7, Ghana_Afr9, Madagascar_Afr11, Madagascar_Afr12, Madagascar_Afr13, Tanzania_Afr15, Tanzania_Afr16, Tanzania_Afr18, Tanzania_Afr20, Tanzania_Afr23, Tanzania_Afr24, Tanzania_Afr26
Tanzania_Afr14, Tanzania_Afr17, Tanzania_Afr21, Tanzania_Afr22, Tanzania_Afr25
B
Ghana_Afr1, Ghana_Afr2, Ghana_Afr3, Ghana_Afr5, Ghana_Afr6, Ghana_Afr7, Ghana_Afr9, Madagascar_Afr11, Madagascar_Afr12, Madagascar_Afr13, Tanzania_Afr17, Tanzania_Afr22, Tanzania_Afr23, Tanzania_Afr25, Tanzania_Afr26
Gabon_Afr33
Gabon_Afr30, Gabon_Afr34, Gabon_Afr35, Tanzania_Afr14, Tanzania_Afr15, Tanzania_Afr16, Tanzania_Afr18, Tanzania_Afr20, Tanzania_Afr21, Tanzania_Afr24
C
Gabon_Afr30, Gabon_Afr33, Gabon_Afr34, Gabon_Afr35, Tanzania_Afr14, Tanzania_Afr15, Tanzania_Afr16, Tanzania_Afr18, Tanzania_Afr20, Tanzania_Afr21, Tanzania_Afr24
Ghana_Afr1, Ghana_Afr2, Ghana_Afr3, Ghana_Afr5, Ghana_Afr6, Ghana_Afr7, Ghana_Afr9, Madagascar_Afr11, Madagascar_Afr12, Madagascar_Afr13, Tanzania_Afr17, Tanzania_Afr22, Tanzania_Afr23, Tanzania_Afr25, Tanzania_Afr26

D

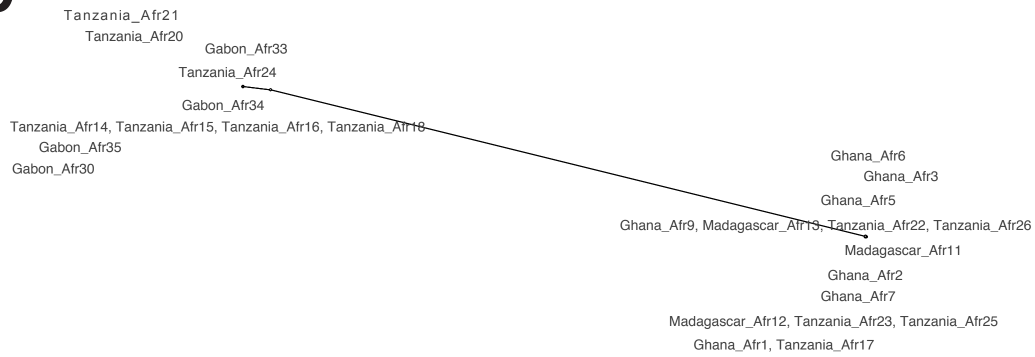
Tanzania_Afr21
Tanzania_Afr20
Gabon_Afr33
Tanzania_Afr24
Gabon_Afr34
Tanzania_Afr14, Tanzania_Afr15, Tanzania_Afr16, Tanzania_Afr18
Gabon_Afr35
Gabon_Afr30
Ghana_Afr6
Ghana_Afr3
Ghana_Afr5
Ghana_Afr9, Madagascar_Afr13, Tanzania_Afr22, Tanzania_Afr26
Madagascar_Afr11
Ghana_Afr2
Ghana_Afr7
Madagascar_Afr12, Tanzania_Afr23, Tanzania_Afr25
Ghana_Afr1, Tanzania_Afr17

E

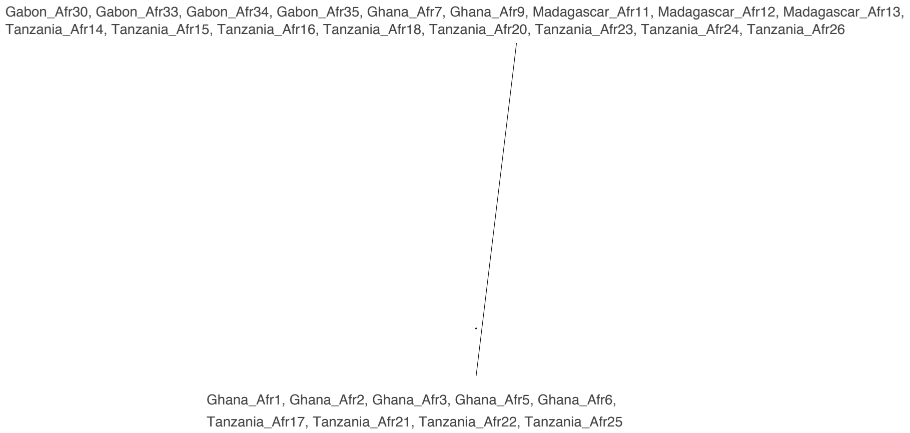
Gabon_Afr30, Gabon_Afr33, Gabon_Afr34, Gabon_Afr35, Ghana_Afr7, Ghana_Afr9, Madagascar_Afr11, Madagascar_Afr12, Madagascar_Afr13,
Tanzania_Afr14, Tanzania_Afr15, Tanzania_Afr16, Tanzania_Afr18, Tanzania_Afr20, Tanzania_Afr23, Tanzania_Afr24, Tanzania_Afr26
Ghana_Afr1, Ghana_Afr2, Ghana_Afr3, Ghana_Afr5, Ghana_Afr6,
Tanzania_Afr17, Tanzania_Afr21, Tanzania_Afr22, Tanzania_Afr25

F

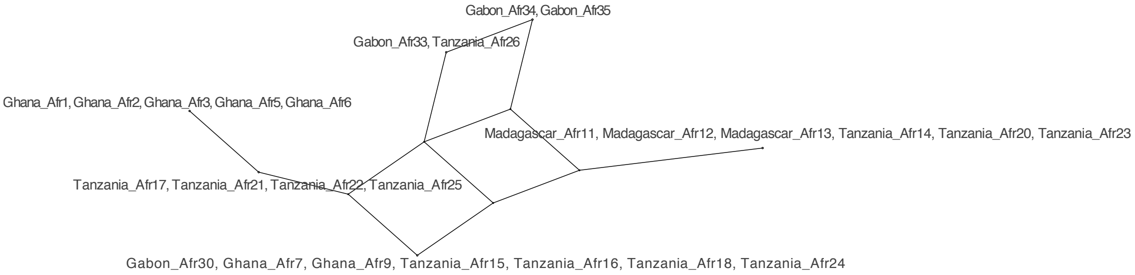
Gabon_Afr34, Gabon_Afr35
Gabon_Afr33, Tanzania_Afr26
Ghana_Afr1, Ghana_Afr2, Ghana_Afr3, Ghana_Afr5, Ghana_Afr6
Madagascar_Afr11, Madagascar_Afr12, Madagascar_Afr13, Tanzania_Afr14, Tanzania_Afr20, Tanzania_Afr23
Tanzania_Afr17, Tanzania_Afr21, Tanzania_Afr22, Tanzania_Afr25
Gabon_Afr30, Ghana_Afr7, Ghana_Afr9, Tanzania_Afr15, Tanzania_Afr16, Tanzania_Afr18, Tanzania_Afr24

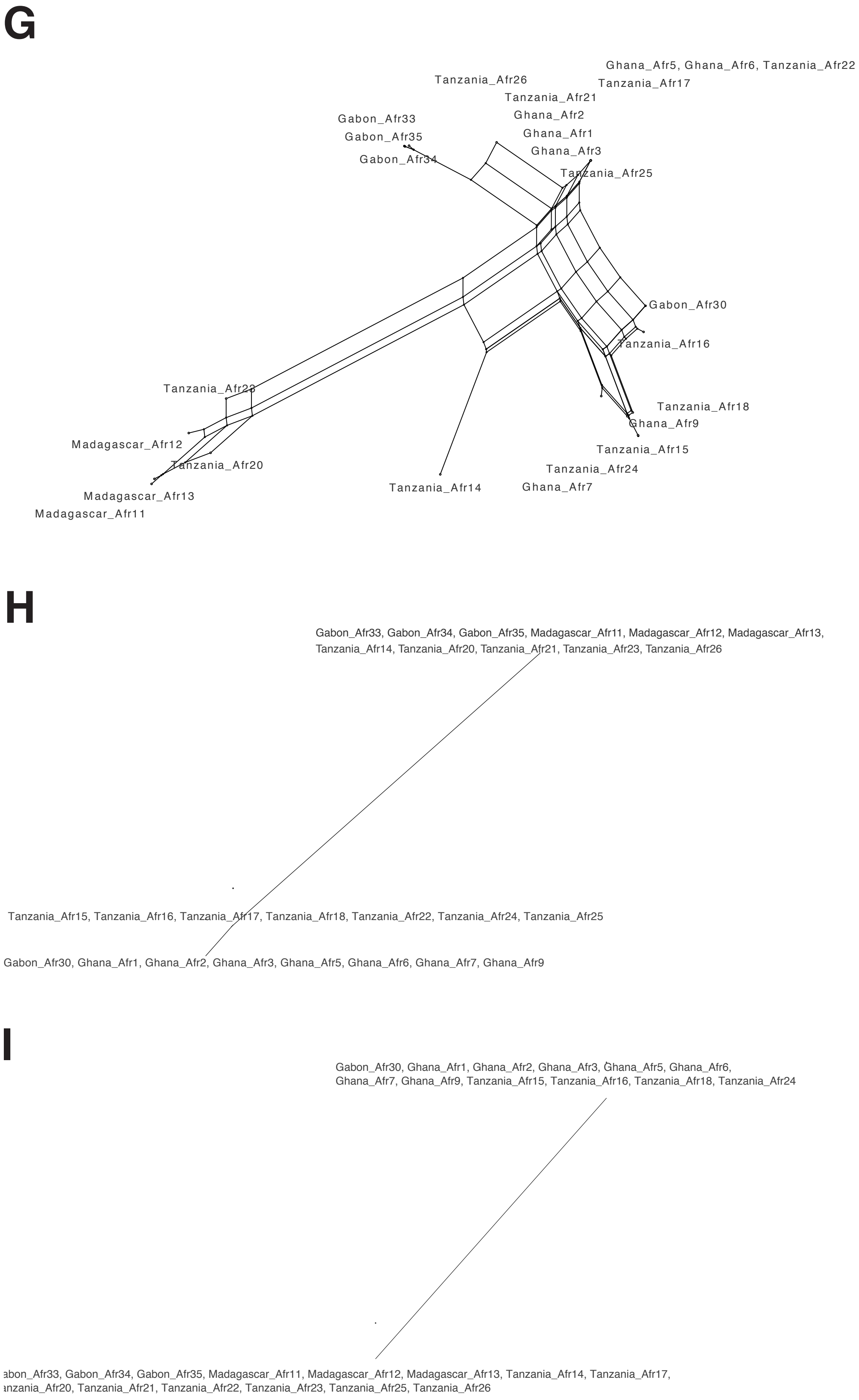
G
Ghana_Afr5, Ghana_Afr6, Tanzania_Afr22
Tanzania_Afr17
Tanzania_Afr26
Tanzania_Afr21
Ghana_Afr2
Ghana_Afr1
Ghana_Afr3
Gabon_Afr33
Gabon_Afr35
Gabon_Afr34
Tanzania_Afr25
Gabon_Afr30
Tanzania_Afr16
Tanzania_Afr23
Tanzania_Afr18
Ghana_Afr9
Madagascar_Afr12
Tanzania_Afr15
Tanzania_Afr20
Tanzania_Afr24
Tanzania_Afr14
Ghana_Afr7
Madagascar_Afr13
Madagascar_Afr11
H
Gabon_Afr33, Gabon_Afr34, Gabon_Afr35, Madagascar_Afr11, Madagascar_Afr12, Madagascar_Afr13,
Tanzania_Afr14, Tanzania_Afr20, Tanzania_Afr21, Tanzania_Afr23, Tanzania_Afr26
Tanzania_Afr15, Tanzania_Afr16, Tanzania_Afr17, Tanzania_Afr18, Tanzania_Afr22, Tanzania_Afr24, Tanzania_Afr25
Gabon_Afr30, Ghana_Afr1, Ghana_Afr2, Ghana_Afr3, Ghana_Afr5, Ghana_Afr6, Ghana_Afr7, Ghana_Afr9
I
Gabon_Afr30, Ghana_Afr1, Ghana_Afr2, Ghana_Afr3, Ghana_Afr5, Ghana_Afr6,
Ghana_Afr7, Ghana_Afr9, Tanzania_Afr15, Tanzania_Afr16, Tanzania_Afr18, Tanzania_Afr24
abon_Afr33, Gabon_Afr34, Gabon_Afr35, Madagascar_Afr11, Madagascar_Afr12, Madagascar_Afr13, Tanzania_Afr14, Tanzania_Afr17,
anzania_Afr20, Tanzania_Afr21, Tanzania_Afr22, Tanzania_Afr23, Tanzania_Afr25, Tanzania_Afr26

Figure S3 – STRUCTURE [9] plot representing the percentage of shared ancestry among the four African *C. hominis* population for K=2 to K=10.

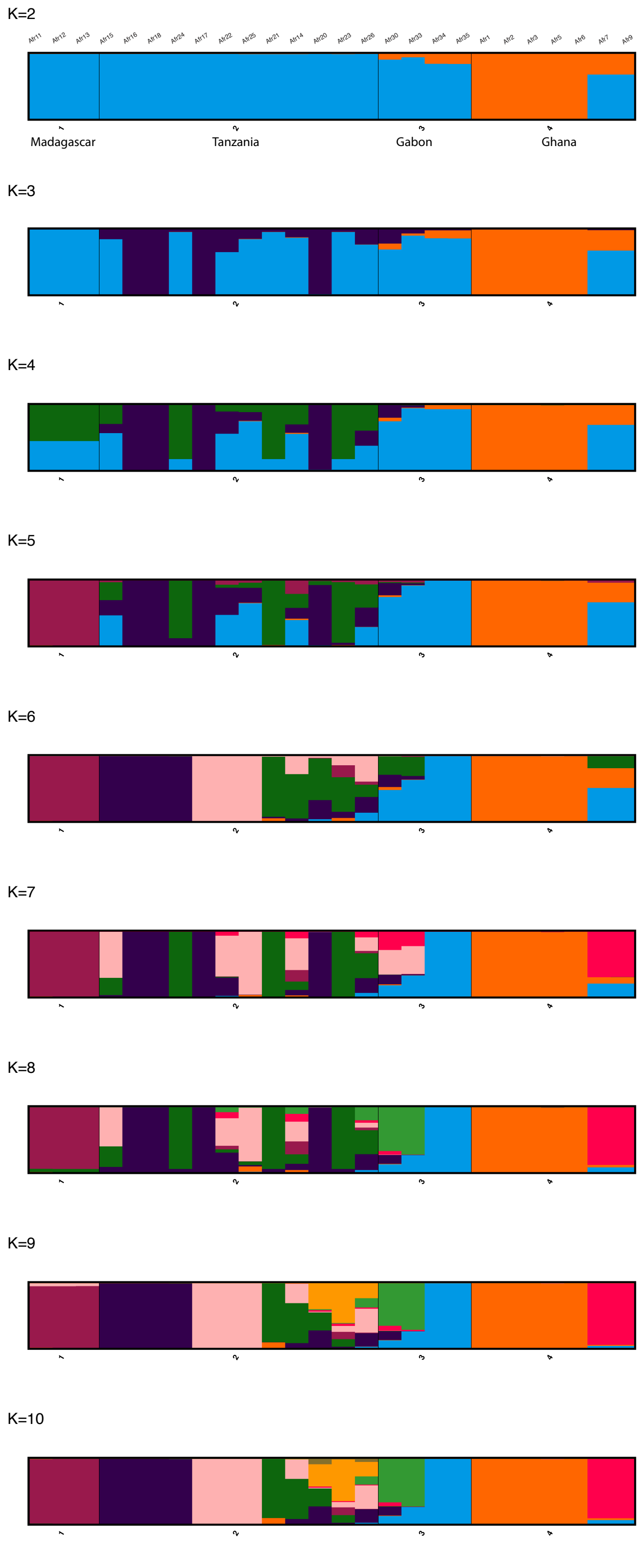

Figure S4 – Plot showing the correlation between genetic diversification (Dxy) and the geographical distance (in km) among the sampled locations (Ghana, Gabon, Madagascar and Tanzania). In total, 58% of the genetic diversification was explained by the geographic distance between sampling locations, and the relationship between both variables appears to be non-linear (Quadratic Regression: $F_{2,63} = 46.77$, $r^2 = 0.58$, linear component: p =1.551e-10, quadratic component: p = 3.543e-13).

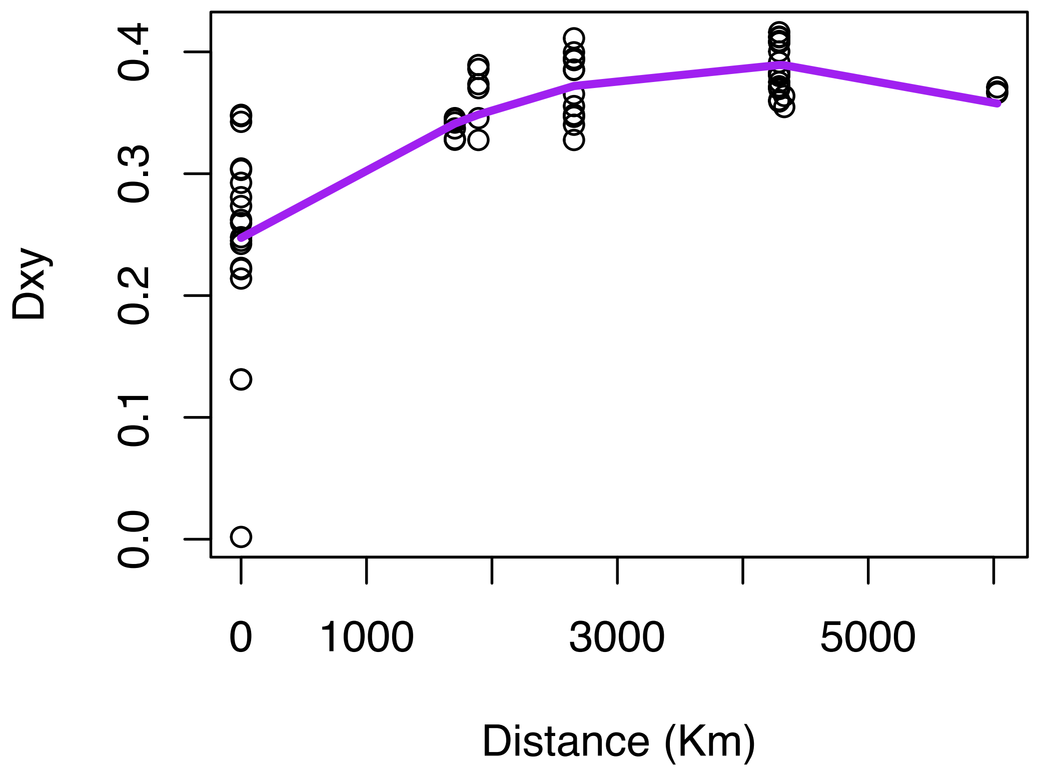

Figure S5 – Density of SNPs (red bars) and INDELs (blue bars) at the whole genome level (Panel A) and in coding sequences (Panel B).

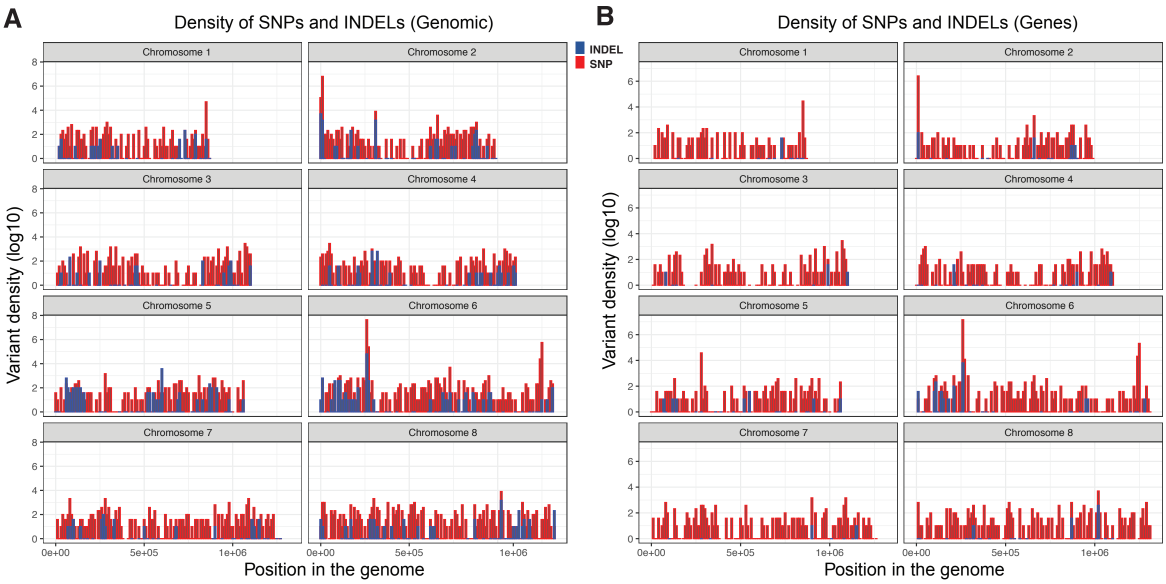

Figure S6 – Proportion the outlier genes encoding for extracellular proteins (solid circles) and signal-peptide containing proteins (open circles). The outlier genes are ranked according to their level of nucleotide diversity (from high to low). The dotted line shows the proportion of extracellular-protein encoding genes in the genome, and the dashed line the proportion of signal-peptide encoding genes in the genome (0.126 and 0.175, respectively).

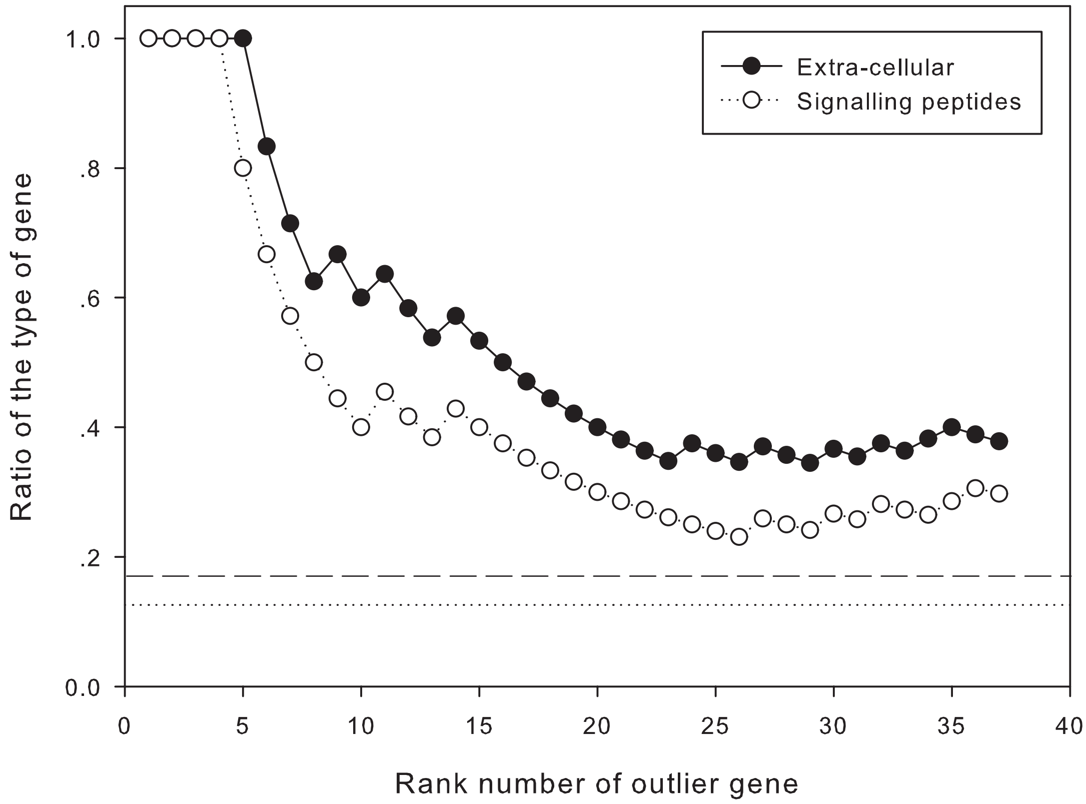

Figure S7. Binomial probabilities of observing equal or more extracellular encoding genes (top), and signal-peptide encoding genes (bottom) in the 37 outlier genes having the highest nucleotide diversity.

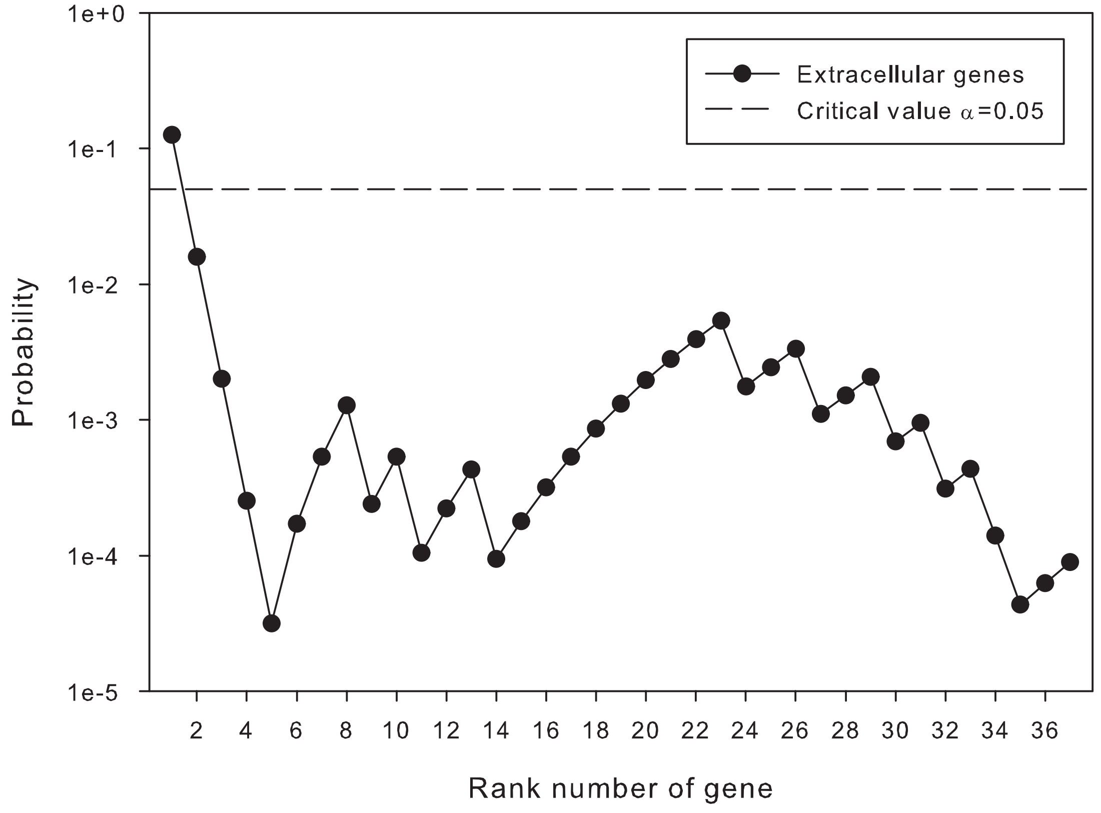

1e+0
1e-1
1e-2
1e-3
1e-4
1e-5
Probability
2 4 6 8 10 12 14 16 18 20 22 24 26 28 30 32 34 36
Rank number of gene
Extracellular genes
Critical value α=0.05

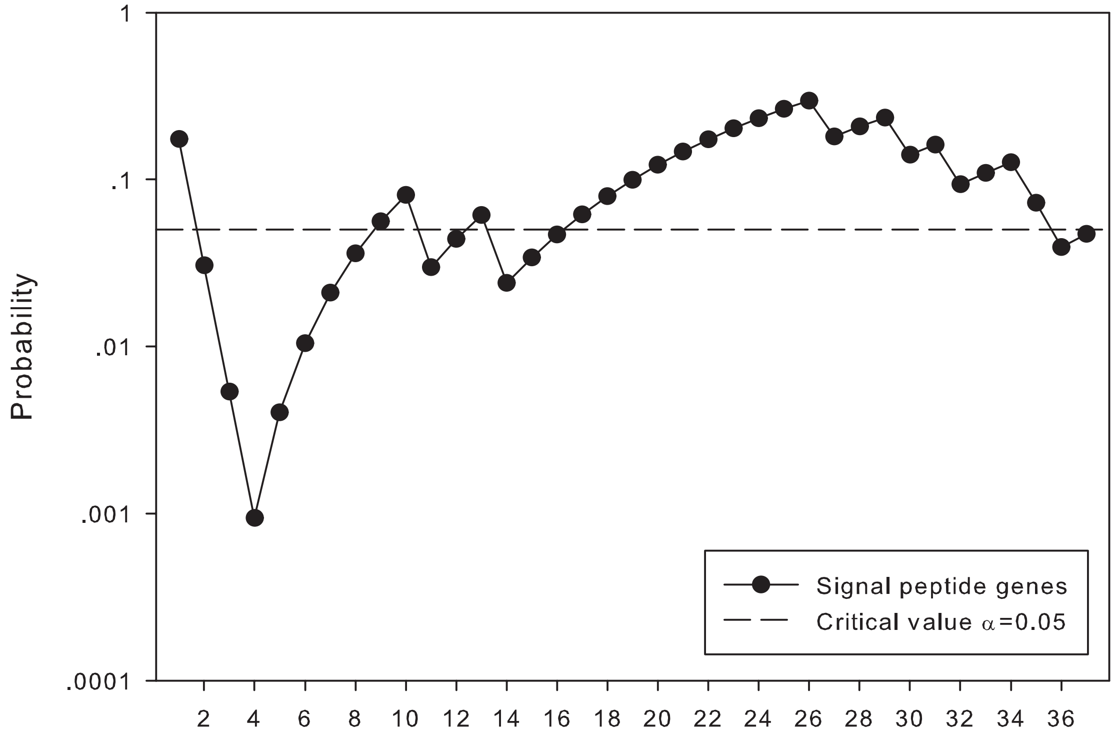

1
.1
.01
.001
.0001
Probability
2 4 6 8 10 12 14 16 18 20 22 24 26 28 30 32 34 36
Rank number of gene
Signal peptide genes
Critical value α=0.05

Figure S8 – Linkage disequilibrium (LD) decay plot of a gene cluster (CHUDEA2_430, CHUDEA2_440 and CHUDEA2_450) on chromosome 2 that encode for mucins in an orthologous species *C. parvum*. Horizontal red line suggests complete linkage within this cluster.

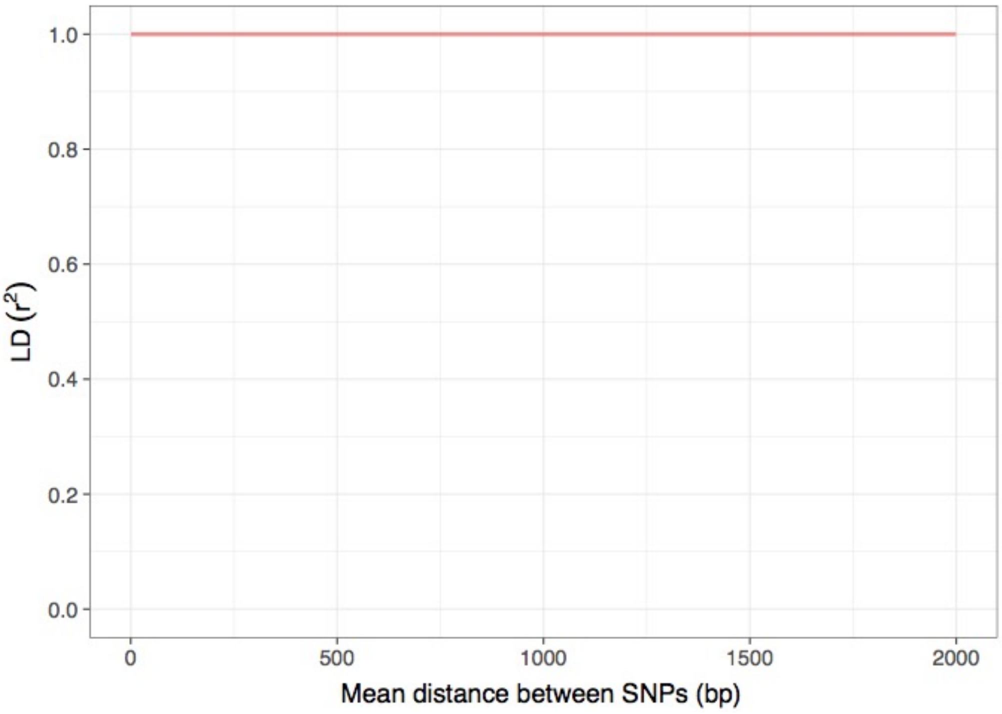

Figure S9 – Haplotypes in African metapopulation. The plot demonstrates multiple sequence alignment of each of **A.** CHUDEA2_430, **B.** CHUDEA2_440 and **C.** CHUDEA2_450 that encodes for mucins in an orthologous species *C. parvum*. All three genes show two haplotypes in African metapopulation. White sites represent consensus while coloured sites represent SNPs.

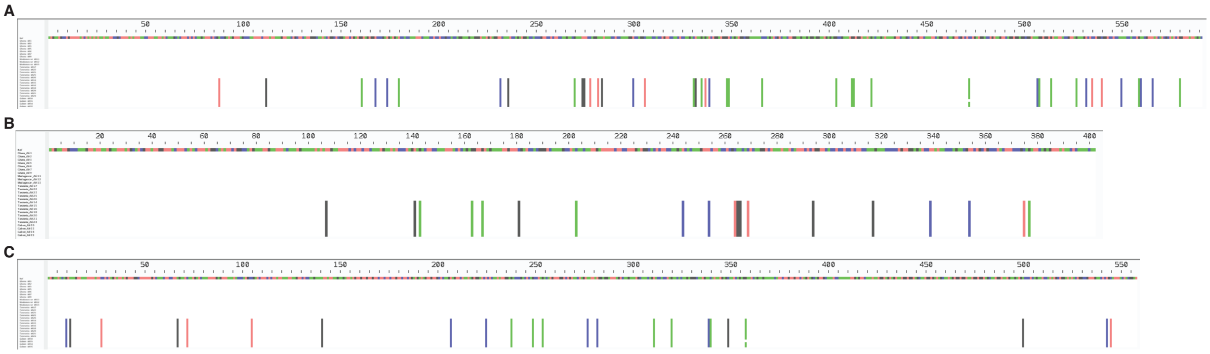

Figure S10 – Closer view of haplotypes in African metapopulation. The plot demonstrates multiple sequence alignment of concatenated SNPs of each of **A.** CHUDEA2_430, **B.** CHUDEA2_440 and **C.** CHUDEA2_450. All three genes show two haplotypes in African metapopulation.

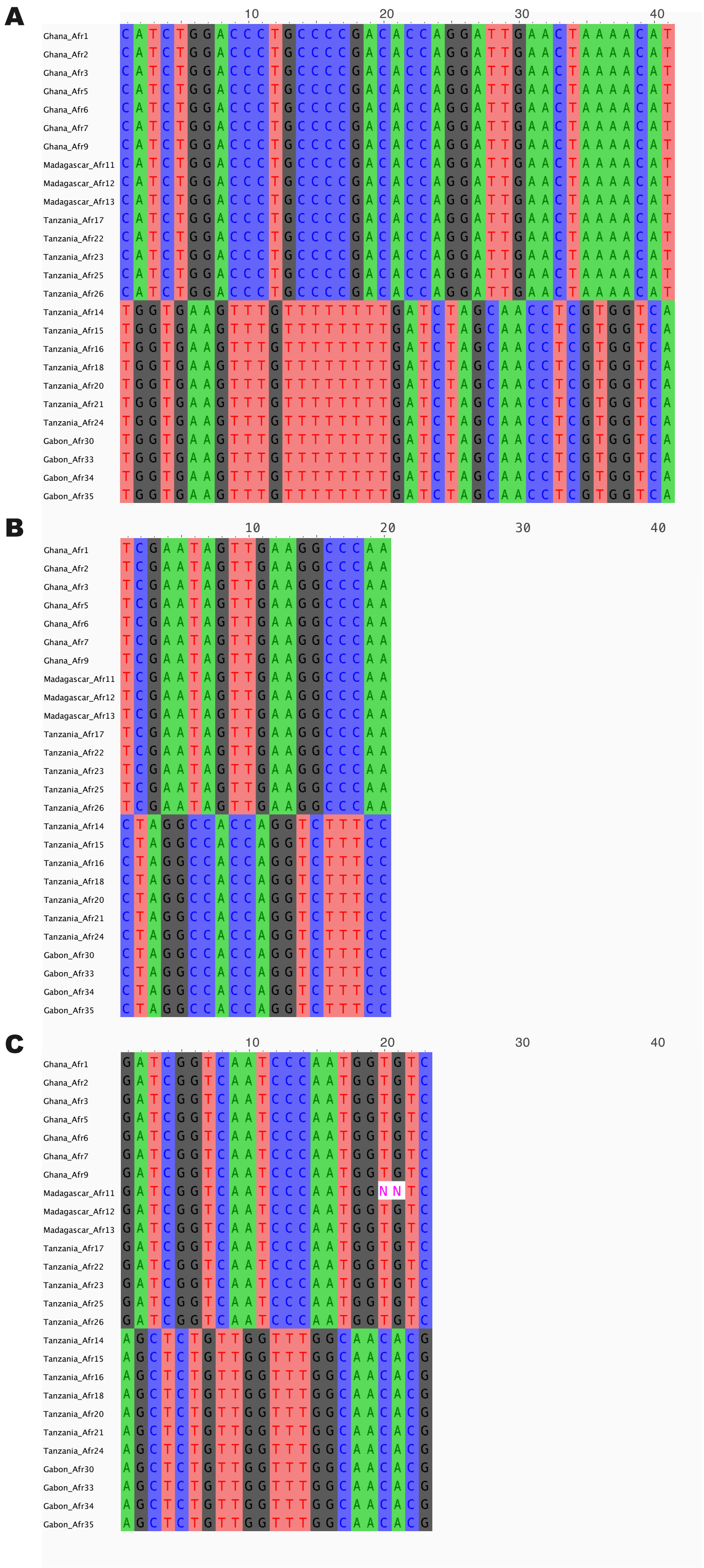
A
10 20 30 40
Ghana_Afr1 CATCTGGACCCTGCCCCGACACCAGGATTGAACTAAAACAT
Ghana_Afr2 CATCTGGACCCTGCCCCGACACCAGGATTGAACTAAAACAT
Ghana_Afr3 CATCTGGACCCTGCCCCGACACCAGGATTGAACTAAAACAT
Ghana_Afr5 CATCTGGACCCTGCCCCGACACCAGGATTGAACTAAAACAT
Ghana_Afr6 CATCTGGACCCTGCCCCGACACCAGGATTGAACTAAAACAT
Ghana_Afr7 CATCTGGACCCTGCCCCGACACCAGGATTGAACTAAAACAT
Ghana_Afr9 CATCTGGACCCTGCCCCGACACCAGGATTGAACTAAAACAT
Madagascar_Afr11 CATCTGGACCCTGCCCCGACACCAGGATTGAACTAAAACAT
Madagascar_Afr12 CATCTGGACCCTGCCCCGACACCAGGATTGAACTAAAACAT
Madagascar_Afr13 CATCTGGACCCTGCCCCGACACCAGGATTGAACTAAAACAT
Tanzania_Afr17 CATCTGGACCCTGCCCCGACACCAGGATTGAACTAAAACAT
Tanzania_Afr22 CATCTGGACCCTGCCCCGACACCAGGATTGAACTAAAACAT
Tanzania_Afr23 CATCTGGACCCTGCCCCGACACCAGGATTGAACTAAAACAT
Tanzania_Afr25 CATCTGGACCCTGCCCCGACACCAGGATTGAACTAAAACAT
Tanzania_Afr26 CATCTGGACCCTGCCCCGACACCAGGATTGAACTAAAACAT
Tanzania_Afr14 TGGTGAAGTTTGTTTTTTTTGATCTAGCAACCTCGTGGTCA
Tanzania_Afr15 TGGTGAAGTTTGTTTTTTTTGATCTAGCAACCTCGTGGTCA
Tanzania_Afr16 TGGTGAAGTTTGTTTTTTTTGATCTAGCAACCTCGTGGTCA
Tanzania_Afr18 TGGTGAAGTTTGTTTTTTTTGATCTAGCAACCTCGTGGTCA
Tanzania_Afr20 TGGTGAAGTTTGTTTTTTTTGATCTAGCAACCTCGTGGTCA
Tanzania_Afr21 TGGTGAAGTTTGTTTTTTTTGATCTAGCAACCTCGTGGTCA
Tanzania_Afr24 TGGTGAAGTTTGTTTTTTTTGATCTAGCAACCTCGTGGTCA
Gabon_Afr30 TGGTGAAGTTTGTTTTTTTTGATCTAGCAACCTCGTGGTCA
Gabon_Afr33 TGGTGAAGTTTGTTTTTTTTGATCTAGCAACCTCGTGGTCA
Gabon_Afr34 TGGTGAAGTTTGTTTTTTTTGATCTAGCAACCTCGTGGTCA
Gabon_Afr35 TGGTGAAGTTTGTTTTTTTTGATCTAGCAACCTCGTGGTCA
B
10 20 30 40
Ghana_Afr1 TCGAATAGTTGAAGGCCCAA
Ghana_Afr2 TCGAATAGTTGAAGGCCCAA
Ghana_Afr3 TCGAATAGTTGAAGGCCCAA
Ghana_Afr5 TCGAATAGTTGAAGGCCCAA
Ghana_Afr6 TCGAATAGTTGAAGGCCCAA
Ghana_Afr7 TCGAATAGTTGAAGGCCCAA
Ghana_Afr9 TCGAATAGTTGAAGGCCCAA
Madagascar_Afr11 TCGAATAGTTGAAGGCCCAA
Madagascar_Afr12 TCGAATAGTTGAAGGCCCAA
Madagascar_Afr13 TCGAATAGTTGAAGGCCCAA
Tanzania_Afr17 TCGAATAGTTGAAGGCCCAA
Tanzania_Afr22 TCGAATAGTTGAAGGCCCAA
Tanzania_Afr23 TCGAATAGTTGAAGGCCCAA
Tanzania_Afr25 TCGAATAGTTGAAGGCCCAA
Tanzania_Afr26 TCGAATAGTTGAAGGCCCAA
Tanzania_Afr14 CTAGGCCACCAGGTCTTTCC
Tanzania_Afr15 CTAGGCCACCAGGTCTTTCC
Tanzania_Afr16 CTAGGCCACCAGGTCTTTCC
Tanzania_Afr18 CTAGGCCACCAGGTCTTTCC
Tanzania_Afr20 CTAGGCCACCAGGTCTTTCC
Tanzania_Afr21 CTAGGCCACCAGGTCTTTCC
Tanzania_Afr24 CTAGGCCACCAGGTCTTTCC
Gabon_Afr30 CTAGGCCACCAGGTCTTTCC
Gabon_Afr33 CTAGGCCACCAGGTCTTTCC
Gabon_Afr34 CTAGGCCACCAGGTCTTTCC
Gabon_Afr35 CTAGGCCACCAGGTCTTTCC
C
10 20 30 40
Ghana_Afr1 GATCGGTCAATCCCAATGGTGTC
Ghana_Afr2 GATCGGTCAATCCCAATGGTGTC
Ghana_Afr3 GATCGGTCAATCCCAATGGTGTC
Ghana_Afr5 GATCGGTCAATCCCAATGGTGTC
Ghana_Afr6 GATCGGTCAATCCCAATGGTGTC
Ghana_Afr7 GATCGGTCAATCCCAATGGTGTC
Ghana_Afr9 GATCGGTCAATCCCAATGGTGTC
Madagascar_Afr11 GATCGGTCAATCCCAATGGNNTC
Madagascar_Afr12 GATCGGTCAATCCCAATGGTGTC
Madagascar_Afr13 GATCGGTCAATCCCAATGGTGTC
Tanzania_Afr17 GATCGGTCAATCCCAATGGTGTC
Tanzania_Afr22 GATCGGTCAATCCCAATGGTGTC
Tanzania_Afr23 GATCGGTCAATCCCAATGGTGTC
Tanzania_Afr25 GATCGGTCAATCCCAATGGTGTC
Tanzania_Afr26 GATCGGTCAATCCCAATGGTGTC
Tanzania_Afr14 AGCTCTGTTGGTTTGGCAACACG
Tanzania_Afr15 AGCTCTGTTGGTTTGGCAACACG
Tanzania_Afr16 AGCTCTGTTGGTTTGGCAACACG
Tanzania_Afr18 AGCTCTGTTGGTTTGGCAACACG
Tanzania_Afr20 AGCTCTGTTGGTTTGGCAACACG
Tanzania_Afr21 AGCTCTGTTGGTTTGGCAACACG
Tanzania_Afr24 AGCTCTGTTGGTTTGGCAACACG
Gabon_Afr30 AGCTCTGTTGGTTTGGCAACACG
Gabon_Afr33 AGCTCTGTTGGTTTGGCAACACG
Gabon_Afr34 AGCTCTGTTGGTTTGGCAACACG
Gabon_Afr35 AGCTCTGTTGGTTTGGCAACACG

Figure S11 – Boxplot showing the distribution of the number of SNPs in each of the 27 *C. hominis* African isolates. The Tanzanian Afr29 isolate was identified as a *C. hominis*/*C. parvum* mixed infection, and therefore excluded. The Madagascar Afr10 isolate was removed from subsequent analyses as it represents an outlier.

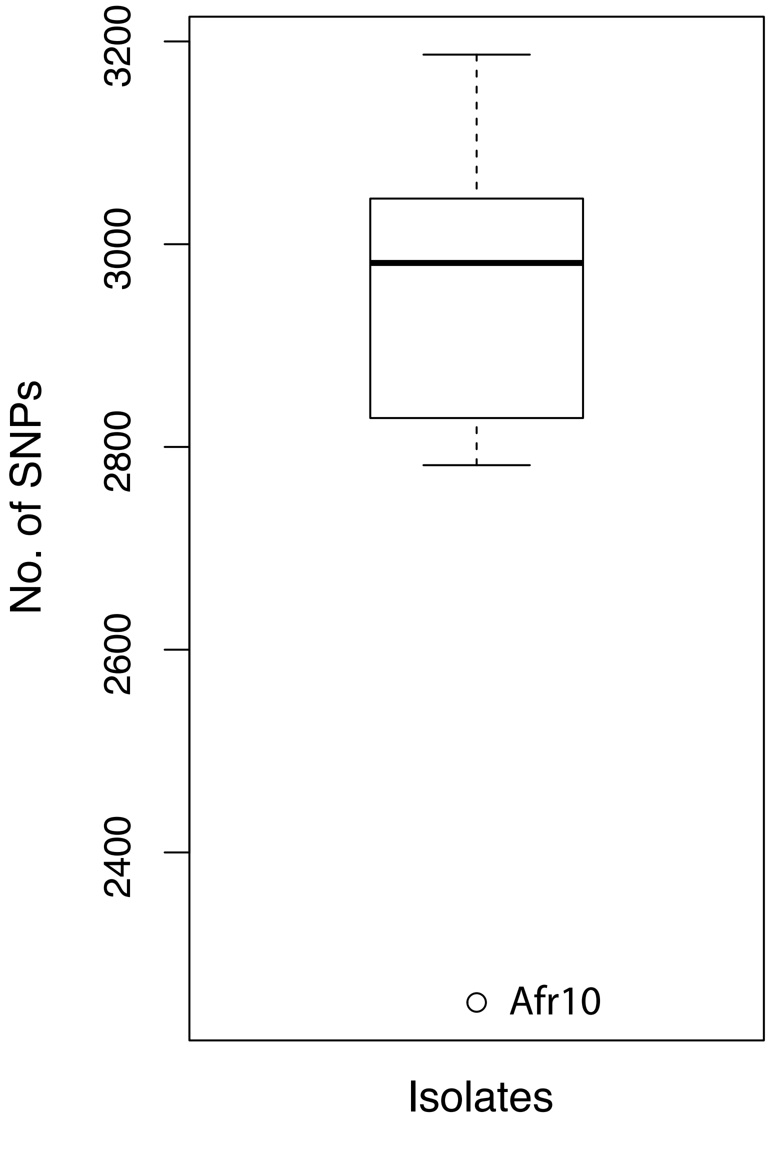

References

1. **Van der Auwera GA, Carneiro MO, Hartl C, Poplin R, Del Angel G et al.** From FastQ data to high confidence variant calls: the Genome Analysis Toolkit best practices pipeline. *Current protocols in bioinformatics* 2013;43:11.10.11-33.
2. **O'Connor RM, Burns PB, Ha-Ngoc T, Scarpato K, Khan W et al.** Polymorphic mucin antigens CpMuc4 and CpMuc5 are integral to Cryptosporidium parvum infection in vitro. *Eukaryot Cell* 2009;8(4):461-469.
3. **Gilchrist CA, Cotton JA, Burkey C, Arju T, Gilmartin A et al.** Genetic Diversity of Cryptosporidium hominis in a Bangladeshi Community as Revealed by Whole-Genome Sequencing. *The Journal of infectious diseases* 2018;218(2):259-264.
4. **Widmer G, Sullivan S**. Genomics and population biology of Cryptosporidium species. *Parasite immunology* 2012;34(2-3):61-71.
5. **Guo Y, Tang K, Rowe LA, Li N, Roellig DM et al.** Comparative genomic analysis reveals occurrence of genetic recombination in virulent Cryptosporidium hominis subtypes and telomeric gene duplications in Cryptosporidium parvum. *BMC genomics* 2015;16(1):320.
6. **Nguyen LT, Schmidt HA, von Haeseler A, Minh BQ**. IQ-TREE: a fast and effective stochastic algorithm for estimating maximum-likelihood phylogenies. *Molecular biology and evolution* 2015;32(1):268-274.
7. **Stecher G, Tamura K, Kumar S**. Molecular Evolutionary Genetics Analysis (MEGA) for macOS. *Molecular biology and evolution* 2020;37(4):1237-1239.
8. **Huson DH, Bryant D**. Application of phylogenetic networks in evolutionary studies. *Molecular biology and evolution* 2006;23(2):254-267.
9. **Pritchard JK, Stephens M, Donnelly P**. Inference of population structure using multilocus genotype data. *Genetics* 2000;155(2):945-959.

S1 Table: **List of significant ($p<0.05$) recombination events obtained by RDP4 analyses of chromosome 6 (LN877952) for 26 African isolates.**

Event	Begin	End	Recombinant Sequence(s)	Minor Parental Sequence(s)	Major Parental Sequence(s)	RDP	GENECONV	Bootscan	Maxchi	Chimaera
1	261808	263589	^Ghana_Afr9	Gabon_Afr34	Unknown (Madagascar_Afr12)	4.52E-15	8.07E-14	2.25E-05	9.58E-09	2.34E-07
			Ghana_Afr7	Gabon_Afr35	Unknown (Madagascar_Afr11)					
			Tanzania_Afr15		Unknown (Madagascar_Afr13)					
			Tanzania_Afr18		Unknown (Tanzania_Afr23)					
2	266739	288394	^Ghana_Afr9	Madagascar_Afr12	Unknown (Gabon_Afr34)	2.77E-14	1.15E-11	NS	3.56E-09	3.10E-04
			Ghana_Afr1	Madagascar_Afr11	Unknown (Gabon_Afr35)					
			Ghana_Afr2	Madagascar_Afr13						
			Ghana_Afr3	Tanzania_Afr14						
			Ghana_Afr5	Tanzania_Afr15						
			Ghana_Afr6	Tanzania_Afr16						
			Ghana_Afr7	Tanzania_Afr17						
				Tanzania_Afr18						
				Tanzania_Afr20						
				Tanzania_Afr21						
				Tanzania_Afr22						
				Tanzania_Afr23						
				Tanzania_Afr24						
				Tanzania_Afr25						
3	262818	263576	^Tanzania_Afr14	Gabon_Afr34	Unknown (Madagascar_Afr12)	2.68E-07	6.80E-05	3.82E-02	1.46E-05	3.66E-05
				Gabon_Afr33	Unknown (Madagascar_Afr11)					
				Gabon_Afr35	Unknown (Madagascar_Afr13)					
					Unknown (Tanzania_Afr20)					
					Unknown (Tanzania_Afr23)					

4	263845	264166	^Tanzania_Afr14	Unknown (Ghana_Afr9)<br>Unknown (Gabon_Afr30)<br>Unknown (Ghana_Afr1)<br>Unknown (Ghana_Afr2)<br>Unknown (Ghana_Afr3)<br>Unknown (Ghana_Afr5)<br>Unknown (Ghana_Afr6)<br>Unknown (Ghana_Afr7)<br>Unknown (Tanzania_Afr15)<br>Unknown (Tanzania_Afr16)<br>Unknown (Tanzania_Afr17)<br>Unknown (Tanzania_Afr18)<br>Unknown (Tanzania_Afr22)<br>Unknown (Tanzania_Afr24)<br>Unknown (Tanzania_Afr25)	Madagascar_Afr12<br>Madagascar_Afr11<br>Madagascar_Afr13<br>Tanzania_Afr17<br>Tanzania_Afr20<br>Tanzania_Afr21<br>Tanzania_Afr22<br>Tanzania_Afr23<br>Tanzania_Afr25	7.28E-06	4.71E-07	NS	9.90E-03	7.49E-05
5	72010	105777*	^Gabon_Afr34<br>Gabon_Afr30<br>Gabon_Afr33<br>Gabon_Afr35	Ghana_Afr9<br>Gabon_Afr30<br>Ghana_Afr7<br>Madagascar_Afr11<br>Madagascar_Afr12<br>Madagascar_Afr13	Unknown (Tanzania_Afr14)<br>Unknown (Tanzania_Afr17)<br>Unknown (Tanzania_Afr22)<br>Unknown (Tanzania_Afr25)	5.27E-06	4.95E-07	1.62E-03	2.62E-03	9.42E-04

				Tanzania_Afr23						
6	103931*	140972	^Ghana_Afr9	Madagascar_Afr12	Unknown (Tanzania_Afr14)	3.27E-08	4.99E-06	NS	2.37E-02	9.42E-04
			Ghana_Afr7	Madagascar_Afr11						
				Madagascar_Afr13						
				Tanzania_Afr23						
7	1187021	1246980*	^Gabon_Afr34	Madagascar_Afr12	Unknown (Ghana_Afr9)	3.58E-12	4.02E-18	1.11E-20	2.24E-11	1.10E-06
			Gabon_Afr33	Gabon_Afr33	Unknown (Gabon_Afr30)					
			Gabon_Afr35	Madagascar_Afr11	Unknown (Ghana_Afr1)					
			Tanzania_Afr14	Madagascar_Afr13	Unknown (Ghana_Afr2)					
			Tanzania_Afr20	Tanzania_Afr14	Unknown (Ghana_Afr3)					
			Tanzania_Afr21	Tanzania_Afr17	Unknown (Ghana_Afr5)					
			Tanzania_Afr23	Tanzania_Afr20	Unknown (Ghana_Afr6)					
			Tanzania_Afr26	Tanzania_Afr21	Unknown (Ghana_Afr7)					
				Tanzania_Afr22						
				Tanzania_Afr25						
8	1246990*	1279726	^Gabon_Afr34	Madagascar_Afr12	Unknown (Ghana_Afr9)	5.07E-05	1.26E-03	1.62E-07	1.87E-06	5.89E-07
			Gabon_Afr33	Tanzania_Afr14						
			Gabon_Afr35							
			Tanzania_Afr14							
			Tanzania_Afr17							
			Tanzania_Afr22							
			Tanzania_Afr25							
			Tanzania_Afr26							
9	264563	265935*	^Tanzania_Afr14	Madagascar_Afr12	Ghana_Afr9	8.84E-09	4.77E-04	4.17E-06	1.77E-06	1.11E-07
			Gabon_Afr30							
			Ghana_Afr1							
			Ghana_Afr2							
			Ghana_Afr3							

Ghana_Afr5
Ghana_Afr6
Tanzania_Afr15
Tanzania_Afr16
Tanzania_Afr17
Tanzania_Afr18
Tanzania_Afr21
Tanzania_Afr22
Tanzania_Afr24
Tanzania_Afr25

Table Key:

*= The actual breakpoint position is undetermined (it was most likely overprinted by a subsequent recombination event).

^= The recombinant sequence may have been misidentified (one of the identified parents might be the recombinant)

Unknown= The sequence listed as unknown was used to infer the existence of a missing parental sequence.

NS = No significant P-value was recorded for this recombination event using this method.

S2 Table. List of significant (p<0.05) recombination events determined by RDP4 analyses of a multiple alignment of chromosome 6 (LN877952) of the four selected African isolates.

Event	Begin	End	Recombinant Sequence(s)	Minor Parental Sequence(s)	Major Parental Sequence(s)	RDP	GENECONV	Bootscan	Maxchi	Chimaera
1	67242	142453	^Madagascar_Afr12<br>Gabon_Afr34	Ghana_Afr9	Tanzania_Afr14	3.27E-08	2.29E-05	7.74E-07	6.53E-04	2.73E-04
2	261529	263504	^Madagascar_Afr12	Unknown (Ghana_Afr9)	Gabon_Afr34	6.24E-07	8.34E-07	1.33E-06	1.67E-09	2.49E-07
3	263506	263734	^Ghana_Afr9	Tanzania_Afr14	Gabon_Afr34	3.00E-05	NS	8.56E-05	7.82E-08	3.89E-05
4	263818	264266	^Madagascar_Afr12	Ghana_Afr9	Tanzania_Afr14	4.08E-06	4.97E-08	3.14E-07	1.00E-05	2.37E-02
5	265862	581836	^Ghana_Afr9	Madagascar_Afr12<br>Tanzania_Afr14	Unknown (Gabon_Afr34)	1.91E-07	3.26E-11	NS	8.10E-06	6.48E-03
6	1186662	1300208	^Gabon_Afr34	Madagascar_Afr12<br>Tanzania_Afr14	Unknown (Ghana_Afr9)	1.60E-19	1.86E-23	3.56E-27	5.04E-06	1.01E-10

Table Key:

^= The recombinant sequence may have been misidentified (one of the identified parents might be the recombinant)

Unknown= The sequence listed as unknown was used to infer the existence of a missing parental sequence.

NS= No significant P-value was recorded for this recombination event using this method.

S3 Table. Pairwise SNP comparison between the four selected isolates from event 1 of Table S2. Values for the recombinant block, and its 3'- and 5'-ends are shown.

**Recombinant Block [67242-142453]**	**Ghana_Afr9**	**Madagascar_Afr12**	**Tanzania_Afr14**	**Gabon_Afr34**
Ghana_Afr9	0	8	21	9
Madagascar_Afr12	-	0	26	11
Tanzania_Afr14	-	-	0	27
Gabon_Afr34	-	-	-	0
**3'end [142454-1317081]**	**Ghana_Afr9**	**Madagascar_Afr12**	**Tanzania_Afr14**	**Gabon_Afr34**
Ghana_Afr9	0	355	317	319
Madagascar_Afr12	-	0	205	267
Tanzania_Afr14	-	-	0	240
Gabon_Afr34	-	-	-	0
**5'end [1-67241]**	**Ghana_Afr9**	**Madagascar_Afr12**	**Tanzania_Afr14**	**Gabon_Afr34**
Ghana_Afr9	0	16	17	19
Madagascar_Afr12	-	0	7	11
Tanzania_Afr14	-	-	0	11
Gabon_Afr34	-	-	-	0

S4 Table. Pairwise SNP comparison between the four selected isolates from event 4 of Supplementary Table 2. Values for the recombinant block, and its 3'- and 5'-ends are shown.

**Recombinant Block [263818-264266]**	**Ghana_Afr9**	**Madagascar_Afr12**	**Tanzania_Afr14**	**Gabon_Afr34**
Ghana_Afr9	0	4	19	6
Madagascar_Afr12	-	0	21	8
Tanzania_Afr14	-	-	0	15
Gabon_Afr34	-	-	-	0
**3'end [264267 -1317081]**	**Ghana_Afr9**	**Madagascar_Afr12**	**Tanzania_Afr14**	**Gabon_Afr34**
Ghana_Afr9	0	229	214	223
Madagascar_Afr12	-	0	97	149
Tanzania_Afr14	-	-	0	141
Gabon_Afr34	-	-	-	0
**5'end [1-263817]**	**Ghana_Afr9**	**Madagascar_Afr12**	**Tanzania_Afr14**	**Gabon_Afr34**
Ghana_Afr9	0	146	122	118
Madagascar_Afr12	-	0	120	132
Tanzania_Afr14	-	-	0	121
Gabon_Afr34	-	-	-	0

S5 Table. Significant recombination events (p<0.05) as detected by RDP4. Event 4 comprises the *gp60* gene (CHUDEA6_1080).

Chromosome	Event	Breakpoint Start	Breakpoint End	Recombinant	Major parent	Minor parent	RDP (p-value)
LN877952	1	67242	142453	Madagascar Afr12	Ghana Afr9	Tanzania Afr14	3.25E-08
LN877952	4	263818	264266	Madagascar_Afr12	Ghana_Afr9	Tanzania_Afr14	4.08E-06

S6 Table. List of 890 most diverse genes that are significantly enriched for signal peptides

Rank	Gene	q-value
1	CHUDEA3_3430	0.00319433
2	CHUDEA2_3360	0.00319433
3	CHUDEA1_370	0.00319433
4	CHUDEA7_4350	0.00319433
5	CHUDEA7_2760	0.00319433
6	CHUDEA6_5180	0.00319433
7	CHUDEA1_650	0.00319433
8	CHUDEA3_3410	0.00319433
9	CHUDEA8_550	0.00319433
10	CHUDEA4_3970	0.00319433
11	CHUDEA8_1230	0.00319433
12	CHUDEA6_5000	0.00319433
13	CHUDEA5_2450	0.00319433
14	CHUDEA5_1940	0.00319433
15	CHUDEA7_3820	0.00319433
16	CHUDEA6_210	0.00319433
17	CHUDEA6_1770	0.00319433
18	CHUDEA3_600	0.00319433
19	CHUDEA8_5270	0.00319433
20	CHUDEA8_4100	0.00319433
21	CHUDEA7_270	0.00319433
22	CHUDEA4_780	0.00319433
23	CHUDEA4_440	0.00319433
24	CHUDEA6_new_01	0.00319433
25	CHUDEA3_2990	0.00319433
26	CHUDEA4_3690	0.00319433
27	CHUDEA2_3710	0.00319433
28	CHUDEA4_1110	0.00319433
29	CHUDEA3_3480	0.00319433
30	CHUDEA3_3000	0.00319433
31	CHUDEA3_3980	0.00319433
32	CHUDEA6_590	0.00319433
33	CHUDEA8_2250	0.00319433
34	CHUDEA8_1700	0.00319433
35	CHUDEA6_3090	0.00319433
36	CHUDEA2_3850	0.00319433
37	CHUDEA8_660	0.00319433
38	CHUDEA5_3300	0.00319433
39	CHUDEA8_3130	0.00319433
40	CHUDEA8_2270	0.00319433
41	CHUDEA7_4990	0.00319433
42	CHUDEA7_460	0.00319433
43	CHUDEA7_1200	0.00319433
44	CHUDEA1_2840	0.00319433
45	CHUDEA4_510	0.00319433

46	CHUDEA1_1430	0.00319433
47	CHUDEA6_780	0.00319433
48	CHUDEA2_2750	0.00319433
49	CHUDEA1_1750	0.00319433
50	CHUDEA3_4090	0.00319433
51	CHUDEA1_1230	0.00319433
52	CHUDEA6_560	0.00319433
53	CHUDEA6_2240	0.00319433
54	CHUDEA7_770	0.00319433
55	CHUDEA7_30	0.00319433
56	CHUDEA6_980	0.00319433
57	CHUDEA5_3960	0.00319433
58	CHUDEA4_2890	0.00319433
59	CHUDEA6_90	0.00319433
60	CHUDEA8_3040	0.00319433
61	CHUDEA6_4200	0.00319433
62	CHUDEA6_1740	0.00319433
63	CHUDEA2_3490	0.00319433
64	CHUDEA2_4220	0.00319433
65	CHUDEA1_1780	0.00319433
66	CHUDEA7_860	0.00319433
67	CHUDEA2_970	0.00319433
68	CHUDEA8_1080	0.00319433
69	CHUDEA1_130	0.00319433
70	CHUDEA1_360	0.00319433
71	CHUDEA5_170	0.00319433
72	CHUDEA7_1270	0.00319433
73	CHUDEA1_670	0.00319433
74	CHUDEA6_1920	0.00319433
75	CHUDEA8_1270	0.00319433
76	CHUDEA8_3260	0.00319433
77	CHUDEA4_new_01	0.00319433
78	CHUDEA5_2960	0.00319433
79	CHUDEA6_2500	0.00319433
80	CHUDEA1_2150	0.00319433
81	CHUDEA4_1860	0.00319433
82	CHUDEA7_710	0.00319433
83	CHUDEA3_2620	0.00319433
84	CHUDEA6_4790	0.00319433
85	CHUDEA7_2530	0.00319433
86	CHUDEA4_2210	0.00319433
87	CHUDEA6_5380	0.00319433
88	CHUDEA8_4780	0.00319433
89	CHUDEA2_2070	0.00319433
90	CHUDEA3_280	0.00319433
91	CHUDEA4_1820	0.00319433
92	CHUDEA4_2850	0.00319433
93	CHUDEA3_2650	0.00319433

94	CHUDEA8_1680	0.00319433
95	CHUDEA7_1360	0.00319433
96	CHUDEA2_4190	0.00319433
97	CHUDEA3_3070	0.00319433
98	CHUDEA4_2390	0.00319433
99	CHUDEA1_2610	0.00319433
100	CHUDEA3_1540	0.00319433
101	CHUDEA4_670	0.00319433
102	CHUDEA4_970	0.00319433
103	CHUDEA7_850	0.00319433
104	CHUDEA2_1250	0.00319433
105	CHUDEA3_410	0.00319433
106	CHUDEA3_2450	0.00319433
107	CHUDEA3_1840	0.00319433
108	CHUDEA7_4690	0.00319433
109	CHUDEA1_450	0.00319433
110	CHUDEA6_4310	0.00319433
111	CHUDEA3_1100	0.00319433
112	CHUDEA7_4530	0.00319433
113	CHUDEA8_2090	0.00319433
114	CHUDEA3_3370	0.00319433
115	CHUDEA8_460	0.00319433
116	CHUDEA4_640	0.00319433
117	CHUDEA5_1490	0.00319433
118	CHUDEA6_4500	0.00319433
119	CHUDEA6_2580	0.00319433
120	CHUDEA4_1010	0.00319433
121	CHUDEA4_2190	0.00319433
122	CHUDEA7_1800	0.00319433
123	CHUDEA7_1760	0.00319433
124	CHUDEA4_3830	0.00319433
125	CHUDEA6_2290	0.00319433
126	CHUDEA7_3320	0.00319433
127	CHUDEA5_130	0.00319433
128	CHUDEA1_2540	0.00319433
129	CHUDEA8_3250	0.00319433
130	CHUDEA4_490	0.00319433
131	CHUDEA8_4620	0.00319433
132	CHUDEA5_420	0.00319433
133	CHUDEA5_1280	0.00319433
134	CHUDEA1_760	0.00319433
135	CHUDEA6_5110	0.00319433
136	CHUDEA3_4200	0.00319433
137	CHUDEA6_4460	0.00319433
138	CHUDEA7_1790	0.00319433
139	CHUDEA2_2340	0.00319433
140	CHUDEA7_5340	0.00319433
141	CHUDEA1_2770	0.00319433

142	CHUDEA5_2830	0.00319433
143	CHUDEA6_2560	0.00319433
144	CHUDEA7_2070	0.00319433
145	CHUDEA5_4250	0.00319433
146	CHUDEA8_1930	0.00319433
147	CHUDEA8_4230	0.00319433
148	CHUDEA8_1450	0.00319433
149	CHUDEA1_750	0.00319433
150	CHUDEA3_2280	0.00319433
151	CHUDEA1_1580	0.00319433
152	CHUDEA5_2410	0.00319433
153	CHUDEA2_2680	0.00319433
154	CHUDEA5_4430	0.00319433
155	CHUDEA4_2470	0.00319433
156	CHUDEA2_10	0.00319433
157	CHUDEA7_420	0.00319433
158	CHUDEA3_4190	0.00319433
159	CHUDEA1_1730	0.00319433
160	CHUDEA8_1320	0.00319433
161	CHUDEA6_1970	0.00319433
162	CHUDEA5_2390	0.00319433
163	CHUDEA5_4230	0.00319433
164	CHUDEA2_2090	0.00319433
165	CHUDEA6_4470	0.00319433
166	CHUDEA5_330	0.00319433
167	CHUDEA5_2860	0.00319433
168	CHUDEA6_4650	0.00319433
169	CHUDEA8_760	0.00319433
170	CHUDEA4_3710	0.00319433
171	CHUDEA2_3550	0.00319433
172	CHUDEA5_40	0.00319433
173	CHUDEA8_2730	0.00319433
174	CHUDEA8_800	0.00319433
175	CHUDEA2_2610	0.00319433
176	CHUDEA1_700	0.00319433
177	CHUDEA7_4230	0.00319433
178	CHUDEA2_2160	0.00319433
179	CHUDEA4_1740	0.00319433
180	CHUDEA8_710	0.00319433
181	CHUDEA4_2320	0.00319433
182	CHUDEA2_700	0.00319433
183	CHUDEA8_4510	0.00319433
184	CHUDEA3_1180	0.00319433
185	CHUDEA8_3530	0.00319433
186	CHUDEA7_3870	0.00319433
187	CHUDEA6_4020	0.00319433
188	CHUDEA1_1450	0.00319433
189	CHUDEA4_3820	0.00319433

190	CHUDEA8_2460	0.00319433
191	CHUDEA5_2460	0.00319433
192	CHUDEA4_3570	0.00319433
193	CHUDEA1_1060	0.00319433
194	CHUDEA2_3430	0.00319433
195	CHUDEA4_3280	0.00319433
196	CHUDEA2_2730	0.00319433
197	CHUDEA5_920	0.00319433
198	CHUDEA6_2100	0.00319433
199	CHUDEA5_3140	0.00319433
200	CHUDEA5_1010	0.00319433
201	CHUDEA4_1590	0.00319433
202	CHUDEA2_2320	0.00319433
203	CHUDEA4_4160	0.00319433
204	CHUDEA6_1790	0.00319433
205	CHUDEA2_90	0.00319433
206	CHUDEA5_4370	0.00319433
207	CHUDEA8_4690	0.00319433
208	CHUDEA8_2650	0.00319433
209	CHUDEA7_920	0.00319433
210	CHUDEA8_4940	0.00319433
211	CHUDEA3_4210	0.00319433
212	CHUDEA1_2300	0.00319433
213	CHUDEA4_4220	0.00319433
214	CHUDEA8_1380	0.00319433
215	CHUDEA7_new_13	0.00319433
216	CHUDEA7_3790	0.00319433
217	CHUDEA6_700	0.00319433
218	CHUDEA1_1560	0.00319433
219	CHUDEA5_3200	0.00319433
220	CHUDEA2_1100	0.00319433
221	CHUDEA6_2450	0.00319433
222	CHUDEA6_1250	0.00319433
223	CHUDEA2_110	0.00319433
224	CHUDEA8_770	0.00319433
225	CHUDEA8_1510	0.00319433
226	CHUDEA8_4170	0.00319433
227	CHUDEA4_4300	0.00319433
228	CHUDEA4_1380	0.00319433
229	CHUDEA1_3820	0.00319433
230	CHUDEA5_2730	0.00319433
231	CHUDEA6_290	0.00319433
232	CHUDEA7_2340	0.00319433
233	CHUDEA6_2180	0.00319433
234	CHUDEA5_2540	0.00319433
235	CHUDEA4_1390	0.00319433
236	CHUDEA6_2300	0.00319433
237	CHUDEA6_490	0.00319433

238	CHUDEA7_3040	0.00319433
239	CHUDEA6_1960	0.00319433
240	CHUDEA7_1710	0.00319433
241	CHUDEA6_3930	0.00319433
242	CHUDEA4_4090	0.00319433
243	CHUDEA5_4360	0.00319433
244	CHUDEA8_1900	0.00319433
245	CHUDEA6_930	0.00319433
246	CHUDEA1_2570	0.00319433
247	CHUDEA5_990	0.00319433
248	CHUDEA5_1200	0.00319433
249	CHUDEA5_550	0.00319433
250	CHUDEA2_1230	0.00319433
251	CHUDEA7_640	0.00319433
252	CHUDEA8_870	0.00319433
253	CHUDEA7_610	0.00319433
254	CHUDEA5_3100	0.00319433
255	CHUDEA6_670	0.00319433
256	CHUDEA7_3030	0.00319433
257	CHUDEA4_4490	0.00319433
258	CHUDEA5_3420	0.00319433
259	CHUDEA4_1690	0.00319433
260	CHUDEA6_3960	0.00319433
261	CHUDEA7_2600	0.00319433
262	CHUDEA6_2490	0.00319433
263	CHUDEA4_3430	0.00319433
264	CHUDEA2_70	0.00319433
265	CHUDEA4_1720	0.00319433
266	CHUDEA2_580	0.00319433
267	CHUDEA7_3570	0.00319433
268	CHUDEA2_2360	0.00319433
269	CHUDEA8_2440	0.00319433
270	CHUDEA5_690	0.00319433
271	CHUDEA4_180	0.00319433
272	CHUDEA8_900	0.00319433
273	CHUDEA8_920	0.00319433
274	CHUDEA8_700	0.00319433
275	CHUDEA1_2240	0.00319433
276	CHUDEA5_1170	0.00319433
277	CHUDEA4_450	0.00319433
278	CHUDEA8_4420	0.00319433
279	CHUDEA8_2960	0.00319433
280	CHUDEA7_5440	0.00319433
281	CHUDEA7_4520	0.00319433
282	CHUDEA4_3270	0.00319433
283	CHUDEA1_3540	0.00319433
284	CHUDEA4_2170	0.00319433
285	CHUDEA7_2720	0.00319433

286	CHUDEA4_4440	0.00319433
287	CHUDEA8_3270	0.00319433
288	CHUDEA6_4840	0.00319433
289	CHUDEA4_860	0.00319433
290	CHUDEA5_150	0.00319433
291	CHUDEA6_2320	0.00319433
292	CHUDEA7_3270	0.00319433
293	CHUDEA2_4270	0.00319433
294	CHUDEA8_4480	0.00319433
295	CHUDEA5_2070	0.00319433
296	CHUDEA6_4560	0.00319433
297	CHUDEA8_3060	0.00319433
298	CHUDEA7_3060	0.00319433
299	CHUDEA8_860	0.00319433
300	CHUDEA1_1220	0.00319433
301	CHUDEA5_1250	0.00319433
302	CHUDEA1_890	0.00319433
303	CHUDEA7_4190	0.00319433
304	CHUDEA2_900	0.00319433
305	CHUDEA6_1280	0.00319433
306	CHUDEA4_3440	0.00319433
307	CHUDEA7_5500	0.00319433
308	CHUDEA6_4410	0.00319433
309	CHUDEA6_730	0.00319433
310	CHUDEA2_680	0.00319433
311	CHUDEA3_1980	0.00319433
312	CHUDEA6_4040	0.00319433
313	CHUDEA6_1180	0.00319433
314	CHUDEA6_2790	0.00319433
315	CHUDEA8_2080	0.00319433
316	CHUDEA3_4150	0.00319433
317	CHUDEA7_3800	0.00319433
318	CHUDEA2_940	0.00319433
319	CHUDEA1_3850	0.00319433
320	CHUDEA8_5210	0.00319433
321	CHUDEA5_610	0.00319433
322	CHUDEA2_3830	0.00319433
323	CHUDEA1_3590	0.00319433
324	CHUDEA2_80	0.00319433
325	CHUDEA4_770	0.00319433
326	CHUDEA8_4030	0.00319433
327	CHUDEA5_1620	0.00319433
328	CHUDEA1_2120	0.00319433
329	CHUDEA3_1790	0.00319433
330	CHUDEA4_2900	0.00319433
331	CHUDEA2_800	0.00319433
332	CHUDEA2_3290	0.00319433
333	CHUDEA8_180	0.00319433

334	CHUDEA3_110	0.00319433
335	CHUDEA8_3430	0.00319433
336	CHUDEA8_640	0.00319433
337	CHUDEA3_1020	0.00319433
338	CHUDEA1_2600	0.00319433
339	CHUDEA1_3790	0.00319433
340	CHUDEA2_2550	0.00319433
341	CHUDEA6_3730	0.00319433
342	CHUDEA5_2930	0.00319433
343	CHUDEA1_640	0.00319433
344	CHUDEA6_320	0.00319433
345	CHUDEA8_3080	0.00319433
346	CHUDEA6_4550	0.00319433
347	CHUDEA6_540	0.00319433
348	CHUDEA2_2980	0.00319433
349	CHUDEA4_3550	0.00319433
350	CHUDEA7_4030	0.00319433
351	CHUDEA6_5270	0.003201599
352	CHUDEA2_3590	0.003207045
353	CHUDEA1_2320	0.003207045
354	CHUDEA6_1900	0.003207045
355	CHUDEA2_3230	0.003207045
356	CHUDEA3_2970	0.003207045
357	CHUDEA6_2070	0.003207045
358	CHUDEA6_1590	0.003207045
359	CHUDEA1_790	0.003207045
360	CHUDEA2_1510	0.003207045
361	CHUDEA4_2240	0.003207045
362	CHUDEA4_3980	0.003207045
363	CHUDEA3_230	0.003207045
364	CHUDEA6_2630	0.003207045
365	CHUDEA7_2870	0.003207045
366	CHUDEA7_1900	0.003231477
367	CHUDEA4_2200	0.003231862
368	CHUDEA6_4290	0.003231862
369	CHUDEA5_2440	0.003242454
370	CHUDEA1_1420	0.003242454
371	CHUDEA8_30	0.003253093
372	CHUDEA6_240	0.003253093
373	CHUDEA5_4390	0.003253099
374	CHUDEA2_300	0.003253099
375	CHUDEA2_3400	0.003253099
376	CHUDEA5_260	0.003253099
377	CHUDEA7_2770	0.003253099
378	CHUDEA6_3160	0.003253099
379	CHUDEA5_730	0.003253396
380	CHUDEA5_3590	0.003274905
381	CHUDEA8_890	0.003274905

382	CHUDEA1_1920	0.003274905
383	CHUDEA6_2370	0.003274905
384	CHUDEA3_2980	0.003274905
385	CHUDEA1_1040	0.003274905
386	CHUDEA7_4560	0.003274905
387	CHUDEA6_3950	0.003281934
388	CHUDEA5_4350	0.003282236
389	CHUDEA8_1340	0.003286013
390	CHUDEA1_3760	0.003286013
391	CHUDEA8_1180	0.003300259
392	CHUDEA6_2140	0.003300259
393	CHUDEA8_1200	0.00331614
394	CHUDEA8_1740	0.003331968
395	CHUDEA3_210	0.003332909
396	CHUDEA7_2380	0.003332909
397	CHUDEA6_160	0.003332909
398	CHUDEA8_1920	0.003336771
399	CHUDEA7_2570	0.003343242
400	CHUDEA4_new_04	0.003343242
401	CHUDEA5_810	0.003343242
402	CHUDEA4_3030	0.003343242
403	CHUDEA8_570	0.003343326
404	CHUDEA8_2410	0.003356491
405	CHUDEA1_3160	0.00340007
406	CHUDEA6_270	0.003416562
407	CHUDEA5_2090	0.003416562
408	CHUDEA1_2700	0.003423518
409	CHUDEA6_2060	0.003423518
410	CHUDEA5_3770	0.003437253
411	CHUDEA1_3840	0.003440043
412	CHUDEA3_4240	0.003440043
413	CHUDEA2_3540	0.003452223
414	CHUDEA6_1430	0.003528968
415	CHUDEA3_200	0.003558223
416	CHUDEA8_1500	0.003577421
417	CHUDEA7_580	0.003603725
418	CHUDEA8_3760	0.003637272
419	CHUDEA4_3310	0.003662632
420	CHUDEA3_530	0.003694952
421	CHUDEA5_3110	0.003704813
422	CHUDEA1_2100	0.003750467
423	CHUDEA8_4010	0.003765355
424	CHUDEA6_3030	0.003765355
425	CHUDEA2_2880	0.003792037
426	CHUDEA5_510	0.003866106
427	CHUDEA5_3370	0.003925179
428	CHUDEA8_90	0.003943137
429	CHUDEA3_2890	0.003944153

430	CHUDEA2_1050	0.003975643
431	CHUDEA1_550	0.004066622
432	CHUDEA6_4170	0.00407207
433	CHUDEA2_560	0.004101743
434	CHUDEA4_1450	0.004118778
435	CHUDEA7_1510	0.004243438
436	CHUDEA8_3680	0.004262264
437	CHUDEA8_4520	0.00426497
438	CHUDEA6_2230	0.004321216
439	CHUDEA4_3450	0.004325965
440	CHUDEA1_3500	0.004358431
441	CHUDEA6_3410	0.0043911
442	CHUDEA8_970	0.004456185
443	CHUDEA4_4270	0.004474749
444	CHUDEA5_670	0.004512003
445	CHUDEA3_160	0.004519526
446	CHUDEA3_3770	0.00455385
447	CHUDEA8_3880	0.00457541
448	CHUDEA1_1720	0.004658108
449	CHUDEA5_4500	0.004678462
450	CHUDEA1_1540	0.004686063
451	CHUDEA6_5350	0.004722109
452	CHUDEA3_2920	0.004789934
453	CHUDEA2_60	0.004868896
454	CHUDEA7_290	0.004868896
455	CHUDEA3_1110	0.004890552
456	CHUDEA6_4140	0.00489603
457	CHUDEA1_3070	0.005069137
458	CHUDEA7_3860	0.005073697
459	CHUDEA6_4690	0.005087054
460	CHUDEA8_4840	0.005087054
461	CHUDEA5_2290	0.005295722
462	CHUDEA8_3110	0.005322469
463	CHUDEA7_4980	0.005349077
464	CHUDEA1_2370	0.005413471
465	CHUDEA1_2750	0.005552858
466	CHUDEA5_3870	0.005581889
467	CHUDEA6_3290	0.005610793
468	CHUDEA1_820	0.005877336
469	CHUDEA2_230	0.005908752
470	CHUDEA4_2150	0.006186367
471	CHUDEA6_2930	0.006220453
472	CHUDEA6_4300	0.006509492
473	CHUDEA7_540	0.00669884
474	CHUDEA4_4480	0.007292504
475	CHUDEA6_30	0.007653064
476	CHUDEA2_730	0.007691359
477	CHUDEA8_3560	0.007797935

478	CHUDEA8_540	0.007816314
479	CHUDEA7_1670	0.007833764
480	CHUDEA3_1660	0.007958079
481	CHUDEA8_1530	0.008000008
482	CHUDEA8_4870	0.008041698
483	CHUDEA3_1500	0.008202203
484	CHUDEA3_1860	0.008207041
485	CHUDEA3_1550	0.008207041
486	CHUDEA8_2520	0.008227901
487	CHUDEA5_3130	0.008298185
488	CHUDEA3_1990	0.00834361
489	CHUDEA6_3360	0.008388825
490	CHUDEA4_3600	0.008557812
491	CHUDEA8_1440	0.008589438
492	CHUDEA2_3030	0.008597391
493	CHUDEA4_1320	0.008597391
494	CHUDEA4_500	0.008621887
495	CHUDEA8_2050	0.008633825
496	CHUDEA2_2560	0.008637204
497	CHUDEA5_1300	0.008665393
498	CHUDEA4_3240	0.008714188
499	CHUDEA7_2410	0.008752007
500	CHUDEA6_2090	0.008752007
501	CHUDEA6_5410	0.008793584
502	CHUDEA5_3850	0.008841673
503	CHUDEA5_3970	0.008886648
504	CHUDEA5_3540	0.008903778
505	CHUDEA7_5090	0.008928711
506	CHUDEA3_2360	0.008928711
507	CHUDEA1_100	0.008931287
508	CHUDEA6_4510	0.008931287
509	CHUDEA1_880	0.008946039
510	CHUDEA4_3590	0.008960466
511	CHUDEA7_5370	0.008980766
512	CHUDEA5_2500	0.009011682
513	CHUDEA3_1140	0.009011682
514	CHUDEA7_3670	0.009034886
515	CHUDEA6_900	0.009049527
516	CHUDEA5_4240	0.009101131
517	CHUDEA7_2850	0.009121942
518	CHUDEA2_2170	0.009198943
519	CHUDEA2_3440	0.009198943
520	CHUDEA1_3520	0.009199054
521	CHUDEA7_3410	0.009199054
522	CHUDEA7_1350	0.009199054
523	CHUDEA6_3260	0.009199054
524	CHUDEA2_450	0.009199054
525	CHUDEA8_40	0.009199054

526	CHUDEA2_1340	0.009199054
527	CHUDEA8_1760	0.009204112
528	CHUDEA2_2940	0.00923979
529	CHUDEA7_3000	0.009298576
530	CHUDEA1_3710	0.009298576
531	CHUDEA2_3150	0.009314395
532	CHUDEA6_2810	0.009314395
533	CHUDEA7_3810	0.009314395
534	CHUDEA5_90	0.009314395
535	CHUDEA8_5310	0.00935599
536	CHUDEA4_1480	0.009393063
537	CHUDEA4_850	0.009393063
538	CHUDEA8_850	0.009429854
539	CHUDEA7_380	0.009429854
540	CHUDEA3_4310	0.009452725
541	CHUDEA5_1710	0.009466342
542	CHUDEA7_2700	0.00949878
543	CHUDEA7_300	0.009502508
544	CHUDEA4_2970	0.009575975
545	CHUDEA5_780	0.009617269
546	CHUDEA8_5330	0.009617269
547	CHUDEA8_790	0.009629749
548	CHUDEA4_4210	0.009629749
549	CHUDEA3_3900	0.009629749
550	CHUDEA7_1140	0.009629749
551	CHUDEA8_3810	0.009664048
552	CHUDEA8_2550	0.009664048
553	CHUDEA3_40	0.009712642
554	CHUDEA6_460	0.009712642
555	CHUDEA5_1900	0.009712642
556	CHUDEA5_3700	0.00974492
557	CHUDEA5_570	0.009750458
558	CHUDEA8_810	0.009784931
559	CHUDEA7_4340	0.009800103
560	CHUDEA8_4750	0.009829117
561	CHUDEA6_1690	0.009874296
562	CHUDEA3_1210	0.009874296
563	CHUDEA5_3560	0.009910947
564	CHUDEA6_3620	0.00994623
565	CHUDEA6_880	0.00994623
566	CHUDEA8_3180	0.00994623
567	CHUDEA8_4020	0.00994623
568	CHUDEA5_1000	0.009990698
569	CHUDEA5_2120	0.009990698
570	CHUDEA7_3740	0.010029977
571	CHUDEA1_1100	0.010029977
572	CHUDEA3_3330	0.010096531
573	CHUDEA7_3190	0.0102983

574	CHUDEA6_3140	0.010321653
575	CHUDEA5_1420	0.010321653
576	CHUDEA4_3320	0.010344038
577	CHUDEA2_520	0.010344038
578	CHUDEA6_2380	0.01035153
579	CHUDEA2_920	0.010359197
580	CHUDEA2_3160	0.010359197
581	CHUDEA3_3880	0.010391039
582	CHUDEA6_830	0.010441411
583	CHUDEA5_3210	0.010691982
584	CHUDEA5_390	0.010712033
585	CHUDEA8_1210	0.010720776
586	CHUDEA3_1580	0.010730385
587	CHUDEA8_2810	0.010730385
588	CHUDEA7_650	0.010730385
589	CHUDEA3_430	0.010777339
590	CHUDEA2_340	0.01084671
591	CHUDEA5_2720	0.01084671
592	CHUDEA2_440	0.01084671
593	CHUDEA2_3870	0.010872936
594	CHUDEA7_3070	0.010953478
595	CHUDEA6_3920	0.011055859
596	CHUDEA3_1890	0.011066973
597	CHUDEA3_990	0.011121673
598	CHUDEA1_2060	0.011141102
599	CHUDEA2_1910	0.01119243
600	CHUDEA8_1250	0.011316361
601	CHUDEA3_1760	0.011357704
602	CHUDEA6_4110	0.011358508
603	CHUDEA8_2060	0.011388548
604	CHUDEA7_4020	0.011405274
605	CHUDEA7_3080	0.011490786
606	CHUDEA4_90	0.01153578
607	CHUDEA6_1700	0.011653979
608	CHUDEA6_3210	0.011710901
609	CHUDEA3_2710	0.011815679
610	CHUDEA7_5050	0.011877119
611	CHUDEA7_3580	0.011877119
612	CHUDEA7_5360	0.011929818
613	CHUDEA1_1690	0.011948543
614	CHUDEA7_1930	0.01198321
615	CHUDEA5_50	0.012113895
616	CHUDEA7_1010	0.012187182
617	CHUDEA3_1160	0.012288087
618	CHUDEA6_4860	0.012334065
619	CHUDEA8_3460	0.012345637
620	CHUDEA4_900	0.012386632
621	CHUDEA7_3050	0.012422101

622	CHUDEA7_2270	0.012425759
623	CHUDEA4_4410	0.012425759
624	CHUDEA3_4340	0.012433005
625	CHUDEA1_2030	0.012471302
626	CHUDEA7_3840	0.012471302
627	CHUDEA8_5220	0.012473449
628	CHUDEA2_960	0.012553124
629	CHUDEA4_4240	0.012660269
630	CHUDEA8_2800	0.012694006
631	CHUDEA8_320	0.012748802
632	CHUDEA2_2260	0.012761078
633	CHUDEA5_4180	0.012761078
634	CHUDEA6_2950	0.012761078
635	CHUDEA4_4500	0.012761078
636	CHUDEA5_910	0.012761078
637	CHUDEA6_650	0.012761078
638	CHUDEA6_1120	0.012761078
639	CHUDEA7_1450	0.012761078
640	CHUDEA8_2490	0.012761078
641	CHUDEA6_1150	0.012834476
642	CHUDEA6_680	0.012834476
643	CHUDEA8_3500	0.012862318
644	CHUDEA1_3450	0.012878565
645	CHUDEA8_1120	0.01292744
646	CHUDEA2_330	0.013104009
647	CHUDEA3_3380	0.013175201
648	CHUDEA4_690	0.013175201
649	CHUDEA1_250	0.013175201
650	CHUDEA2_1000	0.013224591
651	CHUDEA4_2270	0.013347035
652	CHUDEA4_3610	0.013351095
653	CHUDEA7_3900	0.013351095
654	CHUDEA6_3860	0.013351095
655	CHUDEA2_2950	0.013351095
656	CHUDEA6_5420	0.013351095
657	CHUDEA2_2190	0.013351095
658	CHUDEA8_520	0.013356672
659	CHUDEA6_2780	0.013356672
660	CHUDEA7_4110	0.013356672
661	CHUDEA1_1550	0.013669855
662	CHUDEA4_1100	0.013714438
663	CHUDEA3_2490	0.013780668
664	CHUDEA1_3400	0.013803428
665	CHUDEA7_1170	0.013886928
666	CHUDEA4_4360	0.013886928
667	CHUDEA8_3930	0.013962393
668	CHUDEA3_470	0.013962393
669	CHUDEA4_3890	0.013979031

670	CHUDEA4_380	0.013981568
671	CHUDEA5_290	0.013981568
672	CHUDEA6_580	0.013981568
673	CHUDEA3_1360	0.01398804
674	CHUDEA2_2600	0.01398978
675	CHUDEA4_190	0.01398978
676	CHUDEA2_3980	0.014133514
677	CHUDEA3_3710	0.01415806
678	CHUDEA6_2550	0.014241336
679	CHUDEA1_3290	0.014241336
680	CHUDEA6_2640	0.014305472
681	CHUDEA7_4100	0.014305472
682	CHUDEA1_2710	0.014305472
683	CHUDEA5_4170	0.014407623
684	CHUDEA5_2380	0.014576591
685	CHUDEA6_3780	0.014576591
686	CHUDEA7_20	0.014599477
687	CHUDEA7_4290	0.014620648
688	CHUDEA2_2720	0.014621017
689	CHUDEA4_4400	0.014621017
690	CHUDEA8_1910	0.014621017
691	CHUDEA4_4310	0.014621017
692	CHUDEA4_990	0.014621017
693	CHUDEA4_4060	0.014627873
694	CHUDEA8_2260	0.01463075
695	CHUDEA8_4880	0.014644923
696	CHUDEA5_2640	0.014664248
697	CHUDEA2_3460	0.014774738
698	CHUDEA8_3790	0.014916146
699	CHUDEA2_3580	0.014964851
700	CHUDEA7_830	0.015213477
701	CHUDEA7_990	0.015213477
702	CHUDEA4_620	0.015213477
703	CHUDEA4_1160	0.015246076
704	CHUDEA5_1410	0.015323363
705	CHUDEA6_3250	0.015346017
706	CHUDEA8_340	0.015358541
707	CHUDEA8_200	0.015358541
708	CHUDEA8_1970	0.015358541
709	CHUDEA6_1010	0.015369815
710	CHUDEA8_1070	0.015383278
711	CHUDEA7_4310	0.015383278
712	CHUDEA3_2200	0.015594879
713	CHUDEA7_2090	0.015777205
714	CHUDEA2_3620	0.015777205
715	CHUDEA4_1300	0.015831017
716	CHUDEA1_1300	0.01594471
717	CHUDEA4_360	0.015963819

718	CHUDEA8_3360	0.015963819
719	CHUDEA8_4110	0.016059329
720	CHUDEA6_1610	0.016187921
721	CHUDEA8_4730	0.016208193
722	CHUDEA8_3540	0.016224336
723	CHUDEA4_1400	0.016236159
724	CHUDEA6_3630	0.016273932
725	CHUDEA7_4830	0.016323007
726	CHUDEA5_3800	0.01633271
727	CHUDEA2_1370	0.016355096
728	CHUDEA1_1160	0.016459557
729	CHUDEA7_2170	0.016553675
730	CHUDEA1_3030	0.016553675
731	CHUDEA4_4350	0.016683174
732	CHUDEA3_4270	0.016738326
733	CHUDEA6_2250	0.016848863
734	CHUDEA6_1070	0.01697329
735	CHUDEA8_3660	0.01697329
736	CHUDEA3_1050	0.01697329
737	CHUDEA6_1950	0.017017139
738	CHUDEA6_1310	0.017017139
739	CHUDEA6_300	0.017032808
740	CHUDEA5_560	0.017032808
741	CHUDEA4_910	0.017032808
742	CHUDEA8_450	0.01710292
743	CHUDEA3_3560	0.017337592
744	CHUDEA4_610	0.017337592
745	CHUDEA6_2130	0.017474733
746	CHUDEA5_400	0.017634982
747	CHUDEA1_1090	0.017709815
748	CHUDEA7_4920	0.017938023
749	CHUDEA3_380	0.017962453
750	CHUDEA8_5150	0.017994523
751	CHUDEA3_540	0.018022137
752	CHUDEA8_4910	0.018035403
753	CHUDEA4_2110	0.018035403
754	CHUDEA3_3590	0.018184457
755	CHUDEA5_2200	0.018195731
756	CHUDEA3_4070	0.0182723
757	CHUDEA6_2680	0.018294047
758	CHUDEA8_1980	0.018322082
759	CHUDEA8_2320	0.018373104
760	CHUDEA4_110	0.018781397
761	CHUDEA4_2350	0.018955637
762	CHUDEA1_810	0.018968351
763	CHUDEA4_4290	0.019096061
764	CHUDEA7_950	0.019096061
765	CHUDEA6_4920	0.019130193

766	CHUDEA1_3360	0.019182148
767	CHUDEA5_3730	0.019192992
768	CHUDEA4_1370	0.019278285
769	CHUDEA7_5290	0.01968274
770	CHUDEA5_2420	0.01968274
771	CHUDEA3_1480	0.01968274
772	CHUDEA6_5430	0.019808205
773	CHUDEA7_new_01	0.019827984
774	CHUDEA3_new_07	0.019975154
775	CHUDEA2_3090	0.019975154
776	CHUDEA1_560	0.020056344
777	CHUDEA8_2950	0.020056344
778	CHUDEA4_1570	0.020098114
779	CHUDEA2_3900	0.020248282
780	CHUDEA2_1960	0.020645348
781	CHUDEA7_1120	0.020645348
782	CHUDEA4_820	0.020677379
783	CHUDEA8_100	0.021027408
784	CHUDEA6_2920	0.021142217
785	CHUDEA8_1460	0.021248833
786	CHUDEA4_new_07	0.021303994
787	CHUDEA7_1260	0.021338274
788	CHUDEA7_4090	0.021492223
789	CHUDEA4_3990	0.021659303
790	CHUDEA5_1880	0.02167285
791	CHUDEA1_2290	0.022123437
792	CHUDEA1_420	0.022251875
793	CHUDEA8_2530	0.022333872
794	CHUDEA4_4320	0.022502785
795	CHUDEA5_3050	0.02253818
796	CHUDEA2_3300	0.022678186
797	CHUDEA3_3690	0.022797798
798	CHUDEA8_750	0.023436579
799	CHUDEA8_1240	0.023436579
800	CHUDEA1_690	0.023823917
801	CHUDEA7_4840	0.023917309
802	CHUDEA8_4860	0.023967962
803	CHUDEA8_2700	0.023967962
804	CHUDEA2_1550	0.023968629
805	CHUDEA3_1700	0.024126247
806	CHUDEA6_2860	0.024549451
807	CHUDEA6_500	0.024640105
808	CHUDEA8_840	0.025280024
809	CHUDEA7_4620	0.025299642
810	CHUDEA6_1510	0.025313035
811	CHUDEA8_5380	0.025489708
812	CHUDEA5_2880	0.025736533
813	CHUDEA8_4000	0.026582588

814	CHUDEA7_390	0.026582588
815	CHUDEA7_3100	0.027067767
816	CHUDEA7_3180	0.027069518
817	CHUDEA3_4250	0.027263773
818	CHUDEA8_1610	0.027893512
819	CHUDEA4_2830	0.027971483
820	CHUDEA3_1750	0.028104031
821	CHUDEA2_270	0.028349595
822	CHUDEA3_3850	0.028690426
823	CHUDEA8_4770	0.029224213
824	CHUDEA8_2000	0.029416867
825	CHUDEA7_3720	0.029450959
826	CHUDEA6_4800	0.029714421
827	CHUDEA6_5260	0.030351282
828	CHUDEA5_3910	0.030385935
829	CHUDEA8_50	0.030534059
830	CHUDEA4_60	0.030677163
831	CHUDEA1_830	0.030845726
832	CHUDEA5_new_02	0.031092805
833	CHUDEA3_new_02	0.03240713
834	CHUDEA5_2080	0.0324827
835	CHUDEA2_3800	0.032535131
836	CHUDEA8_4740	0.032598136
837	CHUDEA6_710	0.032657818
838	CHUDEA6_5310	0.033207102
839	CHUDEA7_1130	0.034105919
840	CHUDEA2_4010	0.034139534
841	CHUDEA6_4980	0.034139534
842	CHUDEA7_890	0.034397158
843	CHUDEA5_3670	0.034584355
844	CHUDEA4_3630	0.034984284
845	CHUDEA4_4010	0.035748806
846	CHUDEA2_3570	0.036468067
847	CHUDEA2_3890	0.036683032
848	CHUDEA6_2310	0.036893202
849	CHUDEA7_5420	0.037098178
850	CHUDEA6_640	0.03749561
851	CHUDEA7_1480	0.03858849
852	CHUDEA3_4290	0.038832934
853	CHUDEA8_830	0.039073007
854	CHUDEA6_3910	0.039307055
855	CHUDEA4_810	0.039307055
856	CHUDEA6_4000	0.040058966
857	CHUDEA7_5150	0.040294186
858	CHUDEA4_3530	0.04040043
859	CHUDEA3_1720	0.040649656
860	CHUDEA5_2950	0.041184442
861	CHUDEA4_400	0.041205299

862	CHUDEA2_3630	0.041424201
863	CHUDEA6_5280	0.041424201
864	CHUDEA8_250	0.041778731
865	CHUDEA8_3640	0.042192687
866	CHUDEA6_1080	0.042350598
867	CHUDEA1_3800	0.042350598
868	CHUDEA6_2710	0.042979984
869	CHUDEA2_2960	0.043703258
870	CHUDEA4_1420	0.043723134
871	CHUDEA5_490	0.044019482
872	CHUDEA8_2720	0.044043645
873	CHUDEA4_3950	0.044204975
874	CHUDEA5_10	0.044662457
875	CHUDEA4_1760	0.044884162
876	CHUDEA7_3550	0.045589482
877	CHUDEA7_1330	0.046059001
878	CHUDEA8_2570	0.046062617
879	CHUDEA1_2890	0.046119307
880	CHUDEA6_480	0.046488297
881	CHUDEA7_4600	0.046551814
882	CHUDEA4_2770	0.046850069
883	CHUDEA5_1580	0.047535191
884	CHUDEA6_1440	0.048033652
885	CHUDEA8_4200	0.04809451
886	CHUDEA1_1250	0.048481841
887	CHUDEA2_1500	0.04855409
888	CHUDEA6_1050	0.048784156
889	CHUDEA1_3050	0.048878591
890	CHUDEA3_4160	0.049541206

S7 Table. List of 236 most diverse genes that are significantly enriched for extracellular proteins encoding genes

Rank	Gene	q-value
1	CHUDEA2_450	0.006151826
2	CHUDEA3_1690	0.006151826
3	CHUDEA7_3400	0.006151826
4	CHUDEA2_1070	0.006151826
5	CHUDEA6_4980	0.006151826
6	CHUDEA6_5280	0.006151826
7	CHUDEA6_1000	0.006151826
8	CHUDEA5_new_05	0.006151826
9	CHUDEA7_230	0.006151826
10	CHUDEA2_3730	0.006151826
11	CHUDEA7_4650	0.006151826
12	CHUDEA4_new_01	0.006151826
13	CHUDEA6_1100	0.006151826
14	CHUDEA2_new_06	0.006151826
15	CHUDEA8_310	0.006151826
16	CHUDEA5_4440	0.006151826
17	CHUDEA8_5290	0.006151826
18	CHUDEA7_new_10	0.006151826
19	CHUDEA6_40	0.006151826
20	CHUDEA7_140	0.006151826
21	CHUDEA7_4890	0.006151826
22	CHUDEA7_340	0.006151826
23	CHUDEA6_1070	0.006151826
24	CHUDEA5_2990	0.006151826
25	CHUDEA6_3150	0.006151826
26	CHUDEA7_3340	0.006151826
27	CHUDEA6_480	0.006151826
28	CHUDEA1_310	0.006151826
29	CHUDEA1_1200	0.006151826
30	CHUDEA4_1270	0.006151826
31	CHUDEA4_1250	0.006151826
32	CHUDEA7_1410	0.006151826
33	CHUDEA6_860	0.006151826
34	CHUDEA5_2270	0.006151826
35	CHUDEA8_1190	0.006151826
36	CHUDEA3_3550	0.006151826
37	CHUDEA6_1800	0.006151826
38	CHUDEA6_2830	0.006151826
39	CHUDEA5_740	0.006151826
40	CHUDEA6_3580	0.006151826
41	CHUDEA1_900	0.006151826
42	CHUDEA8_new_04	0.006151826
43	CHUDEA6_5270	0.006151826
44	CHUDEA3_4180	0.006151826

45	CHUDEA7_1180	0.006151826
46	CHUDEA3_100	0.006151826
47	CHUDEA6_2890	0.006151826
48	CHUDEA2_3910	0.006151826
49	CHUDEA3_3050	0.006151826
50	CHUDEA1_260	0.006151826
51	CHUDEA5_2030	0.006151826
52	CHUDEA4_10	0.006151826
53	CHUDEA1_3650	0.006151826
54	CHUDEA5_4090	0.006151826
55	CHUDEA2_390	0.006151826
56	CHUDEA8_5110	0.006151826
57	CHUDEA5_3030	0.006151826
58	CHUDEA3_350	0.006151826
59	CHUDEA3_3640	0.006151826
60	CHUDEA1_3550	0.006151826
61	CHUDEA4_3780	0.006151826
62	CHUDEA7_350	0.006151826
63	CHUDEA7_1370	0.006151826
64	CHUDEA5_1370	0.006151826
65	CHUDEA4_140	0.006151826
66	CHUDEA5_4160	0.006151826
67	CHUDEA5_3620	0.006151826
68	CHUDEA7_1240	0.006151826
69	CHUDEA2_150	0.006151826
70	CHUDEA8_4120	0.006151826
71	CHUDEA2_320	0.006151826
72	CHUDEA3_3460	0.006151826
73	CHUDEA2_3670	0.006151826
74	CHUDEA6_1170	0.006151826
75	CHUDEA6_2650	0.006151826
76	CHUDEA6_870	0.006151826
77	CHUDEA7_3300	0.006151826
78	CHUDEA6_2460	0.006151826
79	CHUDEA5_3690	0.006151826
80	CHUDEA3_3670	0.006151826
81	CHUDEA7_3700	0.006151826
82	CHUDEA2_3650	0.006151826
83	CHUDEA3_1060	0.006151826
84	CHUDEA2_620	0.006151826
85	CHUDEA8_3200	0.006151826
86	CHUDEA7_2890	0.006151826
87	CHUDEA5_newCHUDeA_03	0.006151826
88	CHUDEA5_2620	0.006151826
89	CHUDEA3_910	0.006151826
90	CHUDEA8_4370	0.006151826
91	CHUDEA3_960	0.006151826
92	CHUDEA8_1160	0.006151826

93	CHUDEA6_new_04	0.006151826
94	CHUDEA7_2660	0.006151826
95	CHUDEA3_570	0.006151826
96	CHUDEA5_1320	0.006151826
97	CHUDEA7_370	0.006151826
98	CHUDEA8_4090	0.006151826
99	CHUDEA7_1150	0.006151826
100	CHUDEA6_2850	0.006151826
101	CHUDEA4_1220	0.006151826
102	CHUDEA6_5260	0.006151826
103	CHUDEA2_540	0.006151826
104	CHUDEA1_2790	0.006151826
105	CHUDEA5_300	0.006151826
106	CHUDEA1_3310	0.006151826
107	CHUDEA5_2850	0.006151826
108	CHUDEA5_1220	0.006151826
109	CHUDEA1_410	0.006151826
110	CHUDEA3_450	0.006151826
111	CHUDEA2_640	0.006151826
112	CHUDEA2_3140	0.006151826
113	CHUDEA8_4660	0.006151826
114	CHUDEA3_1590	0.006151826
115	CHUDEA6_3810	0.006151826
116	CHUDEA1_1110	0.006151826
117	CHUDEA6_840	0.006151826
118	CHUDEA3_1090	0.00622374
119	CHUDEA2_720	0.006388001
120	CHUDEA5_3750	0.00645949
121	CHUDEA1_1370	0.00645949
122	CHUDEA3_2720	0.00645949
123	CHUDEA5_3890	0.00645949
124	CHUDEA5_2150	0.00645949
125	CHUDEA3_1240	0.00645949
126	CHUDEA4_200	0.00645949
127	CHUDEA2_4080	0.00645949
128	CHUDEA1_3810	0.00645949
129	CHUDEA6_3970	0.006607008
130	CHUDEA7_100	0.006635167
131	CHUDEA3_4300	0.006635167
132	CHUDEA3_4260	0.006635167
133	CHUDEA7_2280	0.006635167
134	CHUDEA5_3950	0.006635167
135	CHUDEA5_460	0.006635167
136	CHUDEA2_670	0.006635167
137	CHUDEA2_3810	0.006988645
138	CHUDEA7_4760	0.006988645
139	CHUDEA1_3080	0.006988645
140	CHUDEA4_1560	0.007086255

141	CHUDEA8_240	0.007150006
142	CHUDEA3_1010	0.007223852
143	CHUDEA3_900	0.007322132
144	CHUDEA8_2870	0.007448304
145	CHUDEA8_1280	0.007640132
146	CHUDEA1_3350	0.007640937
147	CHUDEA6_420	0.007650864
148	CHUDEA8_4680	0.007679239
149	CHUDEA2_4210	0.007699805
150	CHUDEA7_4860	0.007911415
151	CHUDEA3_3990	0.007922523
152	CHUDEA8_3520	0.007935457
153	CHUDEA3_3130	0.008119903
154	CHUDEA8_1960	0.008135519
155	CHUDEA3_1350	0.00843919
156	CHUDEA8_new_10	0.00843919
157	CHUDEA8_new_05	0.00843919
158	CHUDEA6_5300	0.008475362
159	CHUDEA6_5500	0.008479459
160	CHUDEA5_3810	0.008479459
161	CHUDEA1_390	0.008518016
162	CHUDEA6_5240	0.008577371
163	CHUDEA5_4380	0.008577371
164	CHUDEA7_1110	0.008752501
165	CHUDEA4_4370	0.008772778
166	CHUDEA7_4820	0.008843811
167	CHUDEA7_3160	0.008993993
168	CHUDEA6_1050	0.009107874
169	CHUDEA4_950	0.009160846
170	CHUDEA4_170	0.009275113
171	CHUDEA4_920	0.009332266
172	CHUDEA3_3910	0.0093815
173	CHUDEA6_3500	0.009599515
174	CHUDEA4_1800	0.009689926
175	CHUDEA8_20	0.009758063
176	CHUDEA7_2050	0.009758063
177	CHUDEA6_3180	0.009821897
178	CHUDEA6_5450	0.009872249
179	CHUDEA6_1060	0.010161154
180	CHUDEA7_3500	0.010188294
181	CHUDEA3_3170	0.01051137
182	CHUDEA8_3820	0.010619745
183	CHUDEA8_2890	0.010675736
184	CHUDEA3_550	0.010992427
185	CHUDEA8_1400	0.011007854
186	CHUDEA7_2210	0.011007854
187	CHUDEA6_1110	0.011007854
188	CHUDEA4_1130	0.011007854

189	CHUDEA7_4870	0.011077993
190	CHUDEA6_3100	0.011077993
191	CHUDEA3_2100	0.011117412
192	CHUDEA8_400	0.011174711
193	CHUDEA2_1210	0.011741482
194	CHUDEA2_1430	0.011741482
195	CHUDEA4_3800	0.011874232
196	CHUDEA5_3090	0.011874232
197	CHUDEA8_2340	0.011874232
198	CHUDEA2_440	0.012107664
199	CHUDEA6_5160	0.012113386
200	CHUDEA3_360	0.012326542
201	CHUDEA2_860	0.01235629
202	CHUDEA7_1380	0.01237237
203	CHUDEA6_3460	0.01257805
204	CHUDEA5_2210	0.01257805
205	CHUDEA4_1660	0.013250773
206	CHUDEA7_5030	0.013576677
207	CHUDEA1_1030	0.013653095
208	CHUDEA7_1310	0.013653095
209	CHUDEA8_2230	0.013974082
210	CHUDEA4_3130	0.014132734
211	CHUDEA7_2020	0.014288275
212	CHUDEA2_3770	0.014429141
213	CHUDEA7_2460	0.014609697
214	CHUDEA6_2200	0.015136538
215	CHUDEA3_340	0.015544721
216	CHUDEA6_1090	0.015575989
217	CHUDEA6_5250	0.015762426
218	CHUDEA5_1180	0.016200486
219	CHUDEA3_2740	0.017564621
220	CHUDEA8_4190	0.018055765
221	CHUDEA7_5250	0.018938603
222	CHUDEA3_4360	0.020493125
223	CHUDEA7_new_11	0.021113966
224	CHUDEA6_180	0.022054762
225	CHUDEA1_2210	0.023819352
226	CHUDEA7_3250	0.025700609
227	CHUDEA1_160	0.0277043
228	CHUDEA8_1630	0.02820708
229	CHUDEA6_2820	0.02970605
230	CHUDEA6_4910	0.031963211
231	CHUDEA5_3610	0.034360452
232	CHUDEA2_1020	0.036904085
233	CHUDEA5_3880	0.039600549
234	CHUDEA8_new_12	0.042456403
235	CHUDEA8_3160	0.045478318
236	CHUDEA3_4280	0.048673068

S8 Table. List of outlier genes with the highest nucleotide diversity (from high to low), their respective π and Tajima's D values, encoded protein characteristics (extracellular and presence of signal peptides), telomeric position and annotations from related organism, *C. parvum*. CHUDEA6_1080 is the ID for the *gp60* gene.

Rank	Chromosome	Gene ID	Nucleotide diversity	Tajima's D	Extracellular protein	Presence of signal peptide	Telomeric position	Product description
1	Chr 2	CHUDEA2_430	0.03458	3.49938	YES	YES	YES	Signal peptide containing protein
2	Chr 6	**CHUDEA6_1080**	0.03296	0.41174	YES	YES	NO	Glycoprotein GP40
3	Chr 2	CHUDEA2_440	0.02476	3.31824	YES	YES	YES	Signal peptide containing protein
4	Chr 6	CHUDEA6_5270	0.02362	3.58588	YES	YES	NO	Uncharacterized protein
5	Chr 2	CHUDEA2_450	0.02036	3.29591	YES	NO	YES	Signal peptide containing protein
6	Chr 6	CHUDEA6_1070	0.00466	1.70814	NO	NO	NO	Uncharacterized protein
7	Chr 6	CHUDEA6_5260	0.00242	2.99399	NO	NO	NO	ABC transporter ATpase with 2 AAA domains
8	Chr 6	CHUDEA6_1050	0.00214	1.83432	NO	NO	NO	Iron-sulfur cluster assembly protein
9	Chr 1	CHUDEA1_900	0.00201	0.33572	YES	NO	NO	Uncharacterized protein
10	Chr 6	CHUDEA6_2850	0.00196	2.36963	NO	NO	NO	Uncharacterized protein
11	Chr 6	CHUDEA6_5280	0.0019	2.99078	YES	YES	NO	Uncharacterized protein
12	Chr 6	CHUDEA6_2830	0.00158	2.0667	NO	NO	NO	Pre-mRNA-splicing factor SF3a complex subunit 2 (Prp11)
13	Chr 3	CHUDEA3_3670	0.00155	-0.26687	NO	NO	NO	Casein kinase II subunit beta
14	Chr 6	CHUDEA6_4980	0.00154	0.33572	YES	YES	NO	Uncharacterized protein
15	Chr 6	CHUDEA6_480	0.00151	2.0589	NO	NO	NO	Uncharacterized protein
16	Chr 5	CHUDEA5_3030	0.00147	1.50585	NO	NO	NO	Uncharacterized protein
17	Chr 4	CHUDEA4_1220	0.00146	1.17952	NO	NO	NO	Ribosomal biogenesis regulatory protein
18	Chr 3	CHUDEA3_1010	0.00145	1.00189	NO	NO	NO	Uncharacterized protein
19	Chr 4	CHUDEA4_170	0.00134	1.08035	NO	NO	NO	Uncharacterized protein
20	Chr 8	CHUDEA8_2340	0.00131	1.50585	NO	NO	NO	Cold-shock DNA-binding domain-containing protein
21	Chr 6	CHUDEA6_1090	0.00128	0.98498	NO	NO	NO	DnaJ domain containing protein
22	Chr 7	CHUDEA7_new_11	0.00126	1.2766	NO	NO	NO	Unspecified product
23	Chr 8	CHUDEA8_1630	0.00123	0.81184	NO	NO	NO	GINS complex subunit Sld5
24	Chr 7	CHUDEA7_4870	0.00122	0.71889	YES	NO	NO	Uncharacterized protein
25	Chr 8	CHUDEA8_2230	0.00119	1.50585	NO	NO	NO	Histidine phosphatase
26	Chr 8	CHUDEA8_4190	0.00117	-0.03791	NO	NO	NO	Uncharacterized protein
27	Chr 8	CHUDEA8_new_10	0.00117	1.13718	YES	YES	NO	Uncharacterized protein
28	Chr 6	CHUDEA6_1060	0.00116	2.2911	NO	NO	NO	Protein with spectrin repeats, CG12008-like
29	Chr 3	CHUDEA3_360	0.00114	1.25764	NO	NO	NO	Uncharacterized protein
30	Chr 1	CHUDEA1_3810	0.00112	-1.57097	YES	YES	YES	Uncharacterized protein

31	Chr 8	CHUDEA8_4680	0.00112	1.05518	NO	NO	NO	Brix domain containing protein
32	Chr 2	CHUDEA2_390	0.00112	1.61222	YES	YES	YES	Uncharacterized protein
33	Chr 7	CHUDEA7_3700	0.00106	1.39947	NO	NO	NO	Uncharacterized protein
34	Chr 8	CHUDEA8_310	0.001	0.83508	YES	NO	NO	Zinc finger SWIM-type domain-containing protein
35	Chr 3	CHUDEA3_1690	0.00099	1.90403	YES	YES	NO	Uncharacterized protein
36	Chr 7	CHUDEA7_3400	0.00099	1.08035	NO	YES	NO	Uncharacterized protein
37	Chr 2	CHUDEA2_1070	0.00098	0.33572	NO	NO	NO	40S ribosomal protein S25

S9 Table. List of a three highly polymorphic genes cluster on chromosome 2, their respective π, Tajima's D values, Ka/Ks ratio, encoded protein characteristics (extracellular, presence of signal peptides and transmembrane domain) and telomeric position.

Gene ID	Nucleotide diversity	Tajima's D	Ka/Ks	Extracellular protein	Presence of signal peptide	Telomeric position	Transmembrane domain
CHUDEA2_430	0.03458	3.49938	1.08106	YES	YES	YES	Yes
CHUDEA2_440	0.02476	3.31824	0.839407	YES	YES	YES	No
CHUDEA2_450	0.02036	3.29591	0.398425	YES	NO	YES	No

S10 Table. Nucleotide variation of three highly polymorphic genes cluster on chromosome 2 (i.e. CHUDEA2_430, CHUDEA2_440 and CHUDEA2_450) within each of the 4 African countries.

**Nucleotide Diversity**	**CHUDEA2_430**	**CHUDEA2_440**	**CHUDEA2_450**
Gabon	0	0	0.001
Ghana	0	0	0
Madagascar	0	0	0
Tanzania	0.036	0.025	0.022
